# Multiple-Strain Malaria Infection and Its Impacts on *Plasmodium falciparum* Resistance to Antimalarial Therapy: A Mathematical Modelling Perspective

**DOI:** 10.1155/2019/9783986

**Published:** 2019-06-11

**Authors:** Titus Okello Orwa, Rachel Waema Mbogo, Livingstone Serwadda Luboobi

**Affiliations:** Institute of Mathematical Sciences, Strathmore University, P.O. Box 59857-00200, Nairobi, Kenya

## Abstract

The emergence of parasite resistance to antimalarial drugs has contributed significantly to global human mortality and morbidity due to malaria infection. The impacts of multiple-strain malarial parasite infection have further generated a lot of scientific interest. In this paper, we demonstrate, using the epidemiological model, the effects of parasite resistance and competition between the strains on the dynamics and control of *Plasmodium falciparum* malaria. The analysed model has a trivial equilibrium point which is locally asymptotically stable when the parasite's effective reproduction number is less than unity. Using contour plots, we observed that the efficacy of antimalarial drugs used, the rate of development of resistance, and the rate of infection by merozoites are the most important parameters in the multiple-strain *P. falciparum* infection and control model. Although the drug-resistant strain is shown to be less fit, the presence of both strains in the human host has a huge impact on the cost and success of antimalarial treatment. To reduce the emergence of resistant strains, it is vital that only effective antimalarial drugs are administered to patients in hospitals, especially in malaria-endemic regions. Our results emphasize the call for regular and strict surveillance on the use and distribution of antimalarial drugs in health facilities in malaria-endemic countries.

## 1. Introduction

The emergence of parasite resistance [1–4] to antimalarial drugs has contributed significantly to human mortality and morbidity due to malaria infection, worldwide [5–7]. A global malaria control strategy of 1992 [8] that advocated for early diagnosis and prompt treatment has been heavily compromised by the emergence of parasite resistance to antimalarial drugs. The evolution of parasite resistance has been described in [9] as an example of a Darwinian evolution. Parasites undergo mutations in their genome in response to the drug-treated human host. These mutations reduce the rate of parasite elimination from the host and increase their survival chances [9]. The most extensively used antimalarial drugs against the deadly *Plasmodium falciparum* malaria are chloroquine (CQ) and sulfadoxine-pyrimethamine (SP) [10, 11]. These drugs are cheap, easily available, and slowly eliminated from the human body [11]. However, the extensive use of CQ and SP has resulted in *P. falciparum* resistance. This has led to global increase in malaria cases and mortality [12]. In response, the World Health Organization (WHO) in 2006 recommended the use of artemisinin-based combination therapies (ACTs) as a first-line treatment for uncomplicated *P. falciparum* malaria [13]. Resistance to ACTs which are currently the standard treatment for *P. falciparum* is likely to cause global health crisis especially in African regions where *P. falciparum* malaria is endemic [11].

The emergence of parasite resistance to malaria therapy dates back to the 19th century. Quinine (1963) was the first-line antimalarial drug against *P. falciparum* [14]. High mortality cases coupled with high parasite resistance led to the introduction of a second drug, chloroquine (CQ), in 1934 [15]. A decade later, CQ was considered the first-line antimalarial drug by several countries until 1957, when the first focus of *P. falciparum* resistance was detected along the Thai-Cambodia border [16]. In Africa, *P. falciparum* resistance to CQ was first discovered among travelers from Kenya to Tanzania [17]. By 1983, CQ resistance had spread to Sudan, Uganda [18], Zambia [19], and Malawi [20]. Unlike Africa, CQ was replaced for the first time with sulfadoxine-pyrimethamine (SP) as a first-line antimalarial drug in Thailand in 1967. Several other countries in Asia and South America followed thereafter [10]. Resistance to SP was, however, reported the same year [21] in the region. In 1988, CQ was replaced for the first time in Africa. KwaZulu-Natal Province of South Africa replaced CQ with SP [22]. In 1993, the Malawian government changed the treatment policy from CQ to SP. Other African countries followed thereafter: Kenya, South Africa, and Botswana (in 1998); Cameroon and Tanzania (in 2001); and Zimbabwe (in 2000) [23]. The effectiveness of SP was equally undermined by resistance. Unlike CQ, *P. falciparum* resistance to SP was mainly attributed to the long half-life of the drug [24]. Confirmed resistance to the artemisinin derivatives was first reported in Cambodia and Mekong regions in 2008 [25].

To leverage on parasite resistance, cost of treatment, and burden of malaria infection to communities and governments, the WHO recommends the use of artemisinin-based combination therapies (ACTs) as the first- and second-line treatment drugs for uncomplicated *P. falciparum* malaria [25]. ACT is a combination of artemisinin derivatives and a partner monotherapy drug. Artemisinin derivatives include artemether, artesunate, and dihydroartemisinin. These derivatives reduce the parasite biomass within the first three days of therapy, while the partner drug, with longer half-life, eliminates the remaining parasites [26]. The WHO currently recommends five different ACTs: (1) artesunate-amodiaquine (AS + AQ), (2) artesunate-mefloquine (AS + MQ), (3) artesunate + sulfadoxine-pyrimethamine (AS + SP), (4) artemether-lumefantrine (AM-LM), and (5) dihydroartemisinin-piperaquine (DHA + PPQ). Additionally, artesunate-pyronaridine may be used in regions where ACT treatment response is low [26]. Access to ACT has been tremendous in the last 8 years, with a recorded increase of 122 million procured treatment courses for the period 2010–2016. However, resistance to currently used ACTs has important public health consequences, especially in the African region, where resistant *P. falciparum* is predominant.

Numerous cross-sectional studies [27, 28] have revealed the possible impacts of multiple strains of *P. falciparum* on the development of resistance to ACTs. In [29] and citations therein, drug-sensitive parasites are shown to strongly suppress the growth and transmission of drug-resistant *P. falciparum* parasites. Although high-transmission settings such as sub-Saharan Africa account for about 90% of all global malaria deaths, resistance to antimalarial drugs has been shown to emerge from low-transmission settings, such as Southeast Asia and South America [29]. Causes of parasite resistance to ACTs are diverse. Historical studies [30, 31] indicate that antimalarial-resistant parasites could emerge from a handful of lineages. It is argued elsewhere [32, 33] that recombination during sexual reproduction in the mosquito vector could be responsible for the delayed appearance of multilocus resistance in high-transmission regions. Moreover, owing to repeated exposure for many years, individuals in high-transmission settings are likely to develop clinical immunity to malaria, leading to stronger selection for resistance [34]. Studies in [29] also support the hypothesis that in-host competition between drug-sensitive and drug-resistant parasites could inhibit the spread of resistance in high-transmission settings. Owing to their integral role in the recent success of global malaria control, the protection of efficacy of ACTs should be a global health priority [35].

Mathematical models of in-host malaria epidemiology and control constitute important tools in guiding strategies for malaria control [36, 37] and the associated financial planning [38]. While some researchers have focussed on probabilistic models [39, 40], others have investigated the effects of drug treatment and resistance development using dynamic models [41, 42]. A deterministic model by Esteva et al. [43] monitored the impact of drug resistance on the transmission dynamics of malaria in a human population. In [29], the impacts of within-host parasite competition are shown to inhibit the spread of resistance [44, 45]. On the contrary, some models [39, 46] have suggested that within-host competition is likely to speed up the spread of resistance in high-transmission settings due to a phenomenon called “competitive release.” In this paper, we provide theoretical insights using mathematical modelling of the impacts of multiple-strain infections on resistance, dynamics, and antimalarial control of *P. falciparum* malaria.

The rest of the paper is organized as follows: In Section 2, we formulate the within-human malaria model that has both the drug-sensitive and drug-resistant *P. falciparum* parasite strains subject to antimalarial therapy. In Section 3, we analyze the model based on epidemiological theorems. Within-host competition between parasite strains and the effects of antimalarial drug efficacy on parasite clearance are discussed in Section 4. Sensitivity analysis and multiple-strain infection and its effects on resistance and malaria dynamics are demonstrated in Section 5. We conclude the paper in Section 6 by emphasizing the need for antimalarial therapy with the potential to eradicate multiple-strain infection due to *P. falciparum*.

## 2. Model Formulation

We present in this paper a deterministic model that describes the within-human-host competition and transmission dynamics of two strains of *P. falciparum* parasites during malaria infection. The compartmental model considers the coinfection and competition between the drug-sensitive (dss) and the drug-resistant (drs) *P. falciparum* strains in the presence of antimalarial therapy. The drs arise presumably from the dss. The rare mechanism here could possibly be due to single point mutation [47]. Both drs and dss initiate immune responses that follow density-dependent kinetics.

Our model is composed of eight compartments: susceptible/healthy/unparasitized erythrocytes (red blood cells) *X*(*t*), parasitized/infected erythrocytes (*Y*_r_(*t*) and *Y*_s_(*t*)), merozoites (*M*_s_(*t*) and *M*_r_(*t*)), gametocytes (*G*_s_(*t*) and *G*_r_(*t*)), and immune cells *W*(*t*). The healthy erythrocytes (RBCs) make up the resource for competition between the drug-resistant and drug-sensitive parasite strains. The infected red blood cells (IRBCs) and different erythrocytic parasite life cycles are categorized based on the strain of the infecting parasite. The merozoites are therefore categorized into drug-sensitive and drug-resistant strains, denoted by *M*_s_(*t*) and *M*_r_(*t*), respectively. The merozoites invade the healthy erythrocytes during the erythrocytic stage, leading to formation of infected erythrocytes. The variable *Y*_s_(*t*) denotes the red blood cells (RBCs) infected with drug-sensitive merozoites, whereas *Y*_r_(*t*) refers to the RBCs infected with drug-resistant merozoites. Similarly, the variables *G*_s_(*t*) and *G*_r_(*t*) represent drug-sensitive and drug-resistant gametocytes, respectively. Owing to saturation in cell and parasite growth, we consider the nonlinear Michaelis–Mented–Monod function described in [48, 49] and used in [50–53] to model the reductive effects of the immune cells on the parasite and infected-cell populations.

The density of the healthy RBCs is increased at the rate *λ*_*x*_ per healthy RBC per unit time from the host's bone marrow, and healthy RBCs die naturally at a rate *μ*_*x*_. Following parasite invasion by free floating merozoites, the healthy erythrocytes get infected by both drug-sensitive and drug-resistant merozoite strains at the rates *β* and *δ*_r_ *β*, respectively. The parameter *δ*_r_ (with 0 < *δ*_r_ < 1) accounts for the reduced fitness (infectiousness) of the resistant parasite strains in relation to the drug-sensitive strains. The destruction of the healthy red blood cells is however limited by the adaptive immune cells *W*. This is represented by the term 1/(1+*γW*), where *γ* is a measure of the efficacy of the immune cells. The equation that governs the evolution of the healthy RBCs is hence given by

(1) $$\begin{array}{c} \frac{dX}{dt}=\lambda_{x}-\mu_{x}X-\frac{\beta X}{1+\gamma W}\left(M_{\text{s}}+\delta_{\text{r}}M_{\text{r}}\right). \end{array}$$

The parasitized erythrocytes are generated through mass action contact (invasion) between the susceptible healthy erythrocytes *X* and the blood floating merozoites (*M*_r_ and *M*_s_). The merozoites subdivide mitotically, within the infected erythrocytes, into thousands of other merozoites, leading to cell burst and emergence of characteristic symptoms of malaria. Additionally, a single infected erythrocyte undergoes hemolysis at the rate *μ*_ys_ to produce *P* secondary merozoites, sustaining the erythrocytic cycle. The drug-sensitive IRBCs (*Y*_s_) burst open to generate more drug-sensitive merozoites or drug-sensitive gametocytes at the rate *σ*_s_. Similar dynamics are observed with the drug-resistant IRBCs, where the drug-resistant gametocytes are generated at the rate *σ*_r_ from IRBCs. Treatment with ACT is assumed to disfranchise the development of the merozoite within the infected erythrocyte. The drug-infested erythrocytes are hence likely to die faster. This is represented by the term (1 − *ω*_s_)^−1^, where 0 < *ω*_s_ < 1 represents the antimalarial-specific treatment efficacy. In this paper and for purposes of illustration and simulations, *ω*_s_ corresponds to the efficacy of artemether-lumefantrine (AL), which is the recommended first-line antimalarial ACT drug for *P. falciparum* infection in Kenya. We assume that no treatment is available for erythrocytes infected with the resistant parasite strains. The time rate of change for *Y*_s_ and *Y*_r_ takes the following form:

(2) $$\begin{array}{c} \frac{dY_{\text{s}}}{dt}=\frac{\beta XM_{\text{s}}}{1+\gamma W}-\frac{k_{\text{y}}Y_{\text{s}}W}{1+aY_{\text{s}}}-\frac{1}{1-\omega_{\text{s}}}\mu_{\text{ys}}Y_{\text{s}}-\sigma_{\text{s}}Y_{\text{s}},\\ \frac{dY_{\text{r}}}{dt}=\frac{\delta_{\text{r}}\beta XM_{\text{r}}}{1+\gamma W}-\frac{k_{\text{y}}Y_{\text{r}}W}{1+aY_{\text{r}}}-\mu_{\text{yr}}Y_{\text{r}}-\sigma_{\text{r}}Y_{\text{r}}. \end{array}$$

The drug-resistant merozoites *M*_r_ and the drug-resistant gametocytes *G*_r_ die naturally at the rates *μ*_mr_ and *μ*_gr_, respectively. It is further assumed that drug-sensitive merozoites *M*_s_ and gametocytes *G*_s_ may develop into drug-resistant merozoites *M*_r_ and gametocytes *G*_r_ at the rates Ψ_1_ and Ψ_2_, respectively. The cost of resistance associated with AL is represented by the parameter *α*_s_. Parasite resistance to antimalarial drugs exacerbates the erythrocytic cycle and increases the cost of treatment [54, 55]. The higher the resistance to antimalarial therapy, the higher the density of malarial parasites in blood. We therefore model this decline in drug effectiveness by rescaling the density of merozoites produced per bursting parasitized erythrocyte *P* by the factor (1 − *α*_s_), where *α*_s_=1 implies no resistance; that is, the ACT is highly effective in eradicating the parasites. If *α*_s_=0 corresponds to maximum resistance, the used ACT drug is least effective in treating the infection. The converse of these descriptions applies to the drug-resistant *P. falciparum* parasite strains. The equations that govern the rate of change of the infected red blood cells and the merozoites take the following form:

(3) $$\begin{array}{c} \frac{dM_{\text{s}}}{dt}=\left(1-\alpha_{\text{s}}\right)P\mu_{\text{ys}}Y_{\text{s}}-\frac{\beta M_{\text{s}}X}{1+\gamma W}-\frac{k_{\text{m}}M_{\text{s}}W}{1+aM_{\text{s}}}-\left(\Psi_{1}+\mu_{\text{ms}}+\zeta \right)M_{\text{s}},\\ \frac{dM_{\text{r}}}{dt}=\left(1-\alpha_{\text{r}}\right)P\mu_{\text{yr}}Y_{\text{r}}+\Psi_{1}M_{\text{s}}-\frac{\delta_{\text{r}}\beta M_{\text{r}}X}{1+\gamma W}-\frac{k_{\text{m}}M_{\text{r}}W}{1+aM_{\text{r}}}-\mu_{\text{mr}}M_{\text{r}},\\ \frac{dG_{\text{s}}}{dt}=\sigma_{\text{s}}Y_{\text{s}}-\frac{k_{\text{g}}WG_{\text{s}}}{1+aG_{\text{s}}}-\left(\Psi_{2}+\mu_{\text{gs}}+\eta \right)G_{\text{s}},\\ \frac{dG_{\text{r}}}{dt}=\sigma_{\text{r}}Y_{\text{r}}+\Psi_{2}G_{\text{s}}-\frac{k_{\text{g}}WG_{\text{r}}}{1+aG_{\text{r}}}-\mu_{\text{gr}}G_{\text{r}}. \end{array}$$

Antimalarial therapy increases the rate of elimination of drug-sensitive merozoites and gametocytes. This is represented by the nonnegative enhancement parameters *ζ* and *η*, respectively.

Although the innate immunity is faster, it is often limited by the on and off rates in its response to invading pathogens [56, 57]. The adaptive immunity, on the contrary, is very slower at the beginning but lasts long enough to ensure no parasite growth in subsequent infections [27]. We assume an immune system that is independent of the invading parasite strain. For purposes of simplicity, we only consider the adaptive immune system, which is mainly composed of the CD8+T cells [58]. We adopt the assumption that the background recruitment of immune cells is constant (at the rate *λ*_w_). Additionally, the production of the immune cells is assumed to be boosted by the infective and infected cells (*G*_r_, *G*_s_), (*M*_r_, *M*_s_), and (*Y*_r_, *Y*_s_) at constant rates *h*_g_, *h*_m_, and *h*_y_, respectively. Circulating gametocytes, infective merozoites, and infected erythrocytes are removed phagocytotically by the immune cells at the rates *k*_g_ *W*, *k*_m_ *W*, and *k*_y_ *W*, respectively. The immune cells also get depleted through natural death at the rate *μ*_w_. The equation for the immune cells takes the following form:

(4) $$\begin{array}{c} \frac{dW}{dt}=\lambda_{\text{w}}+\left\{\frac{h_{\text{g}}\left(G_{\text{s}}+G_{\text{r}}\right)}{G_{\text{s}}+G_{\text{r}}+e_{\text{g}}}+\frac{h_{\text{y}}\left(Y_{\text{s}}+Y_{\text{r}}\right)}{Y_{\text{s}}+Y_{\text{r}}+e_{\text{y}}}+\frac{h_{\text{m}}\left(M_{\text{s}}+M_{\text{r}}\right)}{M_{\text{s}}+M_{\text{r}}+e_{\text{m}}}\right\}W-\mu_{\text{w}}W. \end{array}$$

Following invasion by the merozoites, the IRBCs either produce merozoites or differentiate into gametocytes upon bursting. The total erythrocyte population at any time *t*, denoted by *C*(*t*), is therefore given by

(5) $$\begin{array}{c} C\left(t\right)=X\left(t\right)+Y_{\text{s}}\left(t\right)+Y_{\text{r}}\left(t\right). \end{array}$$

Similarly, the sum total of *P. falciparum* parasites, denoted by *P*(*t*), within the host at any time *t* is described by the following equation:

(6) $$\begin{array}{c} P\left(t\right)=M_{\text{s}}\left(t\right)+M_{\text{r}}\left(t\right)+G_{\text{s}}\left(t\right)+G_{\text{r}}\left(t\right). \end{array}$$

**Figure 1:**
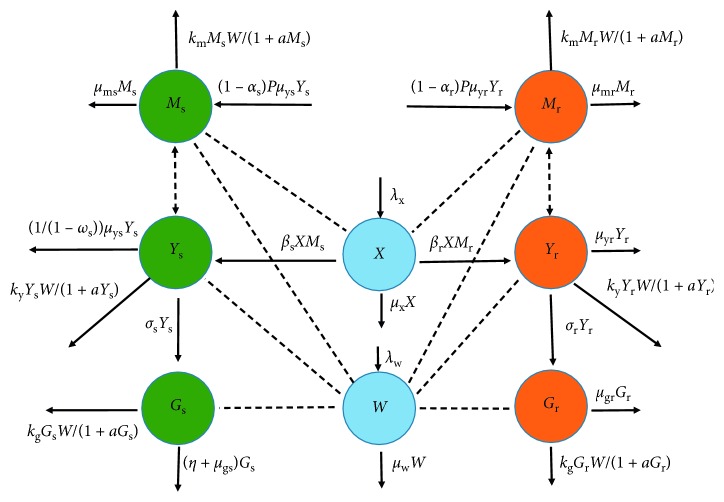
A model flow diagram. Drug-sensitive variables are shown in green colours while the drug-resistant variables are indicated in orange colours. Non-strain-specific variables like susceptible RBCs and immune cells are shown in blue colour. Solid lines indicate the movement of populations from one compartment to another. Dotted lines show possible interactions between the different populations.

The above dynamics can be represented by the schematic diagram in Figure 1. The list of model variables and model parameters is provided in Tables 1 and 2, respectively.

### 2.1. Model Equations

Based on the above model descriptions and schematic diagram shown in Figure 1, the model in this paper consists of the following nonlinear system of ordinary differential equations:

(7) $$\begin{array}{c} \frac{dX}{dt}=\lambda_{x}-\mu_{x}X-\frac{\beta X}{1+\gamma W}\left(M_{\text{s}}+\delta_{\text{r}}M_{r}\right), \end{array}$$

(8) $$\begin{array}{c} \frac{dY_{\text{s}}}{dt}=\frac{\beta XM_{\text{s}}}{1+\gamma W}-\frac{k_{\text{y}}Y_{\text{s}}W}{1+aY_{\text{s}}}-\frac{1}{1-\omega_{\text{s}}}\mu_{\text{ys}}Y_{\text{s}}-\sigma_{\text{s}}Y_{\text{s}}, \end{array}$$

(9) $$\begin{array}{c} \frac{dY_{\text{r}}}{dt}=\frac{\delta_{\text{r}}\beta XM_{\text{r}}}{1+\gamma W}-\frac{k_{\text{y}}Y_{\text{r}}W}{1+aY_{\text{r}}}-\mu_{\text{yr}}Y_{\text{r}}-\sigma_{\text{r}}Y_{\text{r}}, \end{array}$$

(10) $$\begin{array}{c} \frac{dM_{\text{s}}}{dt}=\left(1-\alpha_{\text{s}}\right)P\mu_{\text{ys}}Y_{\text{s}}-\frac{\beta M_{\text{s}}X}{1+\gamma W}-\frac{k_{\text{m}}M_{\text{s}}W}{1+aM_{\text{s}}}-\left(\Psi_{1}+\mu_{\text{ms}}+\zeta \right)M_{\text{s}}, \end{array}$$

(11) $$\begin{array}{c} \frac{dM_{\text{r}}}{dt}=\left(1-\alpha_{\text{r}}\right)P\mu_{\text{yr}}Y_{\text{r}}+\Psi_{1}M_{\text{s}}-\frac{\delta_{\text{r}}\beta M_{\text{r}}X}{1+\gamma W}-\frac{k_{\text{m}}M_{\text{r}}W}{1+aM_{\text{r}}}-\mu_{\text{mr}}M_{\text{r}}, \end{array}$$

(12) $$\begin{array}{c} \frac{dG_{\text{s}}}{dt}=\sigma_{\text{s}}Y_{\text{s}}-\frac{k_{\text{g}}WG_{\text{s}}}{1+aG_{\text{s}}}-\left(\Psi_{2}+\mu_{\text{gs}}+\eta \right)G_{\text{s}}, \end{array}$$

(13) $$\begin{array}{c} \frac{dG_{\text{r}}}{dt}=\sigma_{\text{r}}Y_{\text{r}}+\Psi_{2}G_{\text{s}}-\frac{k_{\text{g}}WG_{\text{r}}}{1+aG_{\text{r}}}-\mu_{\text{gr}}G_{\text{r}}, \end{array}$$

(14) $$\begin{array}{c} \frac{dW}{dt}=\lambda_{\text{w}}+\left\{\frac{h_{\text{g}}\left(G_{\text{s}}+G_{\text{r}}\right)}{G_{\text{s}}+G_{\text{r}}+e_{\text{g}}}+\frac{h_{\text{y}}\left(Y_{\text{s}}+Y_{\text{r}}\right)}{Y_{\text{s}}+Y_{\text{r}}+e_{\text{y}}}+\frac{h_{\text{m}}\left(M_{\text{s}}+M_{\text{r}}\right)}{M_{\text{s}}+M_{\text{r}}+e_{\text{m}}}\right\}W-\mu_{\text{w}}W, \end{array}$$

subject to the following initial conditions:

(15) $$\begin{array}{c} X\left(0\right)>0,\\ Y_{i}\left(0\right)\geq 0,\\ M_{i}\left(0\right)\geq 0,\\ G_{i}\left(0\right)\geq 0,\\ W\left(0\right)>0,\;\text{for}\;\;i=s,r. \end{array}$$

## 3. Model Analysis

### 3.1. Positivity and Uniqueness of Solutions

The consonance between a formulated epidemiological model and its biological reality is key to its usefulness. Given that all the model parameters and variables are nonnegative, it is only sound that the model solutions be nonnegative at any future time *t* ≥ 0 within a given biological space.

**Theorem 1 .**

The region *ℝ*_+_^8^ with solutions of system (7)–(14) is positively invariant under the flow induced by system (7)–(14).

**Proof**

We need to show that every trajectory from the region *ℝ*_+_^8^ will always remain within it. By contradiction, assume ∃*t* ^*∗*^ (where *t* ^*∗*^ refers to time) in the interval [0, *∞*), such that *X*(*t* ^*∗*^)=0, *X*′(*t* ^*∗*^) < 0 but for 0 < *t* < *t* ^*∗*^, *X*(*t*) > 0, and *Y*_*i*_(*t*) > 0, *M*_*i*_(*t*) > 0, *G*_*i*_(*t*) > 0, and *W*_*i*_(*t*) > 0 for *i*={*r*, *s*}. Notice that, at *t*=*t* ^*∗*^, *X*(*t*) is declining from the original zero value. If such an *X* exists, then it should satisfy the differential equation (7). That is,

(16) $$\begin{array}{c} \frac{dX}{dt}=\lambda_{x}-\mu_{x}X\left(t^{*}\right)-\frac{\beta X\left(t^{*}\right)}{1+\gamma W\left(t^{*}\right)}\left(M_{\text{s}}\left(t^{*}\right)+\delta_{\text{r}}M_{\text{r}}\left(t^{*}\right)\right)\\ =\lambda_{x}>0. \end{array}$$

We arrive at a contradiction, i.e., *X*′(*t* ^*∗*^) > 0. This shows the nonexistence of such *t* ^*∗*^. This argument can be extended to all the remaining seven variables (*Y*_s_, *Y*_r_, *M*_s_, *M*_r_, *G*_s_, *G*_r_, *W*). The process of verification is however simpler. We can follow the steps as presented in [59, 60]. Let the total erythrocyte population *C*(*t*) evolve according to the following formulation:

(17) $$\begin{array}{c} \frac{dC}{dt}\leq \lambda_{x}-\mu_{c}C, \end{array}$$

where *μ*_c_=min{*μ*_*x*_, *μ*_ys_, *μ*_yr_}. Similarly, the total density of malarial parasites *P*(*t*) is described by

(18) $$\begin{array}{c} \frac{dP}{dt}\leq P\left\{\left(1-\alpha_{\text{s}}\right)\mu_{\text{ys}}Y_{\text{s}}+\left(1-\alpha_{\text{r}}\right)\mu_{\text{yr}}Y_{\text{r}}\right\}+\sigma_{\text{s}}Y_{\text{s}}+\sigma_{\text{r}}Y_{\text{r}}-\mu_{\text{p}}P, \end{array}$$

where *μ*_p_=min{*μ*_ms_, *μ*_mr_, *μ*_gs_, *μ*_gr_}.

The solutions of equations (14), (17), and (18) are, respectively, given as

(19) $$\begin{array}{c} W\left(t\right)\leq \frac{\lambda_{\text{w}}}{\mu_{\text{w}}}+\left(W\left(0\right)-\frac{\lambda_{\text{w}}}{\mu_{\text{w}}}\right)e^{-\mu_{\text{w}}t},\\ C\left(t\right)\leq \frac{\lambda_{x}}{\mu_{\text{c}}}+\left(C\left(0\right)-\frac{\lambda_{x}}{\mu_{\text{c}}}\right)e^{-\mu_{\text{c}}t},\\ P\left(t\right)\leq \frac{\sigma_{\text{s}}\int_{0}^{t}Y_{\text{s}}\left(t\right)\Delta_{\text{IF}}dt+\sigma_{\text{r}}\int_{0}^{t}Y_{\text{r}}\left(t\right)\Delta_{\text{IF}}dt}{\Delta_{\text{IF}}}+\left(P\left(0\right)-\frac{\left(\sigma_{\text{s}}+\sigma_{\text{r}}\right)\mu_{\text{p}}}{\left(1-\alpha_{\text{s}}\right)\mu_{\text{ys}}+\left(1-\alpha_{\text{r}}\right)\mu_{\text{yr}}}\right)\frac{1}{\Delta_{\text{IF}}}, \end{array}$$

where

(20) $$\begin{array}{c} \Delta_{\text{IF}}=\exp\left\{-\left(\left(1-\alpha_{\text{s}}\right)\mu_{\text{ys}}\int_{0}^{t}Y_{\text{s}}\left(t\right)dt+\left(1-\alpha_{\text{r}}\right)\mu_{\text{yr}}\int_{0}^{t}Y_{\text{r}}\left(t\right)dt\right)-\int_{0}^{t}\mu_{\text{p}}dt\right\}. \end{array}$$

Here, *C*(0)=*X*(0)+*Y*_s_(0)+*Y*_r_(0) and *P*(0)=*M*_s_(0)+*M*_r_(0)+*G*_s_(0)+*G*_r_(0) represent the initial total populations of erythrocytes and malarial parasites, respectively. We observe that all the solutions of equations (14), (17), and (18) remain nonnegative for all future time, *t* ≥ 0. Moreover, the total populations are bounded: 0 ≤ *C*(*t*) ≤ max{*C*(0), (*λ*_*x*_/*μ*_c_)}, 0 ≤ *W*(*t*) ≤ max{*W*(0), *λ*_w_/*μ*_w_} and *P*(*t*) ≤ max(*P*(0), ((*σ*_s_+*σ*_r_)*μ*_p_)/((1 − *α*_s_)*μ*_ys_+(1 − *α*_r_)*μ*_yr_)). Thus, all the state variables of model system (7)–(14) and all their corresponding solutions are nonnegative and bounded in the phase space *φ*, where

(21) $$\begin{array}{c} \varphi =\left[\left(X,Y_{\text{s}},Y_{\text{r}},M_{\text{s}},M_{\text{r}},G_{\text{s}},G_{\text{r}},W\right)\in {\mathbb{R}}_{+}^{8}:C\left(t\right)\leq \max\left\{C\left(0\right),\frac{\lambda_{x}}{\mu_{\text{c}}}\right\},W\left(t\right)\leq \max\left\{W\left(0\right),\frac{\lambda_{\text{w}}}{\mu_{\text{w}}}\right\},P\left(t\right)\leq \max\left(P\left(0\right),\frac{\left(\sigma_{\text{s}}+\sigma_{\text{r}}\right)\mu_{\text{p}}}{\left(1-\alpha_{\text{s}}\right)\mu_{\text{ys}}+\left(1-\alpha_{\text{r}}\right)\mu_{\text{yr}}}\right)\right.. \end{array}$$

It is obvious that *φ* is twice continuously differentiable function. That is, *φ*_*i*_ ∈ *ℂ* ^2^. This is because its components *φ*_*i*_, *i*=1,2,…, 8, are rational functions of state variables that are also continuously differentiable functions. We conclude that the domain *φ* is positively invariant. It is therefore feasible and biological meaningful to study model system (7)–(14).

**Theorem 2 .**

The model system (7)–(14) has a unique solution.

**Proof**

Let **x**=(*X*, *Y*_s_, *Y*_r_, *M*_s_, *M*_r_, *G*_s_, *G*_r_, *W*)^T^ ∈ *ℝ*_+_^8^ so that **x**_1_=*X* and **x**_2_=*Y*_*s*_ as presented in system (7)–(14). Similarly, let **g**(**x**)=(**g**_*i*_(**x**), *i*=1,…,8)^T^ be a vector defined in *ℝ*_+_^8^. The model system (7)–(14) can hence be written as

(22) $$\begin{array}{c} \frac{dx}{dt}=g\left(x\right),\;x\left(0\right)=x_{0}, \end{array}$$

where **x** : [0, *∞*)⟶*ℝ*_+_^8^ denotes a column vector of state variables and **g** : *ℝ*_+_^8^⟶*ℝ*_+_^8^ represents the right-hand side (RHS) of system (7)–(14). The result is as follows.

**Lemma 1 .**

The function **g** is continuously differentiable in **x**.

**Proof**

All the terms in **g** are either linear polynomials or rational functions of nonvanishing polynomials. Since the state variables (*X*, *Y*_s_, *Y*_r_, *M*_s_, *M*_r_, *G*_s_, *G*_r_, *W*) are all continuously differentiable functions of *t*, all the elements of vector **g** are continuously differentiable. Moreover, let *L*(**x**, **n**, *θ*)={**x**+*θ*(**n** − **x**) : 0 ≤ *θ* ≤ 1}. By the mean value theorem,

(23) $$\begin{array}{c} {\left\|g\left(n\right)-g\left(x\right)\right\|}_{\infty }={\left\|g^{\prime }\left(m;n-x\right)\right\|}_{\infty }, \end{array}$$

where **m** ∈ *L*(**x**, **n**, *θ*) denotes the mean value point and **g**′ the directional derivative of the function **g** at **m**. However,

(24) $$\begin{array}{c} {\left\|g^{\prime }\left(m,n-x\right)\right\|}_{\infty }={\left\|\overset{8}{\underset{i=1}{\sum }}\left(▽g_{i}\left(m\right)\cdot \left(n-x\right)\right)e_{i}\right\|}_{\infty }\\ \leq {\left\|\overset{8}{\underset{i=1}{\sum }}\left(▽g_{i}\left(m\right)\right.\right\|}_{\infty }{\left\|n-x\right\|}_{\infty }, \end{array}$$

where *e*_*i*_ is the *i* ^th^ coordinate unit in *ℝ*_+_^8^. We can clearly see that all the partial derivatives of **g** are bounded and that there exists a nonnegative *U* such that

(25) $$\begin{array}{c} {\left\|\overset{8}{\underset{i=1}{\sum }}\left(▽g_{i}\left(m\right)\right.\right\|}_{\infty }\leq U,\;\text{for all}\;\;m\in L. \end{array}$$

Therefore, there exists *U* > 0 such that

(26) $$\begin{array}{c} {\left\|g\left(n\right)-g\left(x\right)\right\|}_{\infty }\leq U{\left\|n-x\right\|}_{\infty }. \end{array}$$

This shows that the function **g** is Lipschitz continuous. Since **g** is Lipschitz continuous, model system (7)–(14) has a unique solution by the uniqueness theorem of Picard [61].

### 3.2. Stability Analysis of the Parasite-Free Equilibrium Point (PFE)

The in-host malaria dynamics are investigated by studying the behaviour of the model at different model equilibrium points. Knowledge on model equilibrium points is useful in deriving parameters that drive the infection to different stability points. The model system (7)–(14) has a parasite-free equilibrium point *𝔼*_0_ given by

(27) $$\begin{array}{c} E_{0}=\left(X_{*},Y_{\text{s}*},Y_{\text{r}*},M_{\text{s}*},M_{\text{r}*},G_{\text{s}*},G_{\text{r}*},W_{*}\right)=\left(\frac{\lambda_{x}}{\mu_{x}},0,0,0,0,0,0,\frac{\lambda_{\text{w}}}{\mu_{\text{w}}}\right). \end{array}$$

Using the next-generation operator method by van den Driessche and Watmough [62] and matrix notations therein, we obtain a nonsingular matrix *Q* showing the terms of transitions from one compartment to the other and a nonnegative matrix *F* of new infection terms as follows:

(28) $$\begin{array}{c} F=\left(\begin{array}{cccccc} 0 & 0 & \frac{\beta \lambda_{x}\mu_{\text{w}}}{\left(\gamma \lambda_{\text{w}}+\mu_{\text{w}}\right)\mu_{x}} & 0 & 0 & 0\\ 0 & 0 & 0 & \frac{\delta_{\text{r}}\beta \lambda_{x}\mu_{\text{w}}}{\left(\gamma \lambda_{\text{w}}+\mu_{\text{w}}\right)\mu_{x}} & 0 & 0\\ 0 & 0 & 0 & 0 & 0 & 0\\ 0 & 0 & 0 & 0 & 0 & 0\\ 0 & 0 & 0 & 0 & 0 & 0\\ 0 & 0 & 0 & 0 & 0 & 0 \end{array}\right), \end{array}$$

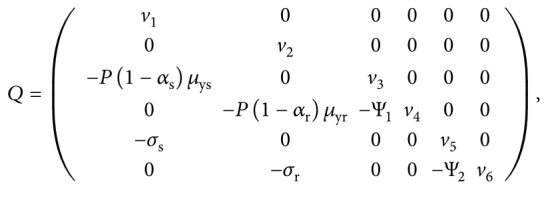
(29)where *v*_1_=((*k*_y_ *λ*_w_/*μ*_w_)+*σ*_s_+(*μ*_ys_/1 − *ω*_s_)), *v*_2_=((*k*_y_ *λ*_w_/*μ*_w_)+*σ*_r_+*μ*_yr_), *v*_4_=(*μ*_mr_+(*k*_m_ *λ*_w_/*μ*_w_)+(*δ*_r_ *βλ*_*x*_ *μ*_w_/(*γλ*_w_+*μ*_w_)*μ*_*x*_)), *v*_3_=(*ζ*+*μ*_ms_+Ψ_1_+(*k*_m_ *λ*_w_/*μ*_w_)+(*βλ*_*x*_ *μ*_w_/(*γλ*_w_+*μ*_w_)*μ*_x_)), and *v*_5_=(*η*+*μ*_gs_+(*k*_g_ *λ*_w_/*μ*_w_)+Ψ_2_), *v*_6_=(*μ*_gr_+(*k*_g_ *λ*_w_/*μ*_w_)).

The effective reproduction number *R*_E_ of model system (7)–(14) associated with the parasite-free equilibrium is the spectral radius of the next-generation matrix FQ^−1^, where

(30) $$\begin{array}{c} Q^{-1}=\left(\begin{array}{cccccc} \frac{1}{v_{1}} & 0 & 0 & 0 & 0 & 0\\ 0 & \frac{1}{v_{2}} & 0 & 0 & 0 & 0\\ \frac{P\left(1-\alpha_{\text{s}}\right)\mu_{\text{ys}}}{v_{1}v_{3}} & 0 & \frac{1}{v_{3}} & 0 & 0 & 0\\ \frac{P\left(1-\alpha_{\text{s}}\right)\mu_{\text{ys}}\Psi_{1}}{v_{1}v_{3}v_{4}} & \frac{P\left(1-\alpha_{\text{r}}\right)\mu_{\text{yr}}}{v_{2}v_{4}} & 0 & \frac{1}{v_{4}} & 0 & 0\\ \sigma_{\text{s}}/v_{1}v_{5} & 0 & 0 & 0 & \frac{1}{v_{5}} & 0\\ \sigma_{\text{s}}\Psi_{2}/v_{1}v_{5}v_{6} & \frac{\sigma_{\text{r}}}{v_{2}v_{6}} & 0 & 0 & \Psi_{2}/v_{5}v_{6} & \frac{1}{v_{6}} \end{array}\right). \end{array}$$

It follows that

(31) $$\begin{array}{c} R_{\text{E}}=\rho \left({\text{FQ}}^{-1}\right)=\text{max}\left\{R_{\text{s}},R_{\text{r}}\right\}, \end{array}$$

where

(32) $$\begin{array}{c} R_{\text{s}}=\frac{P\left(1-\alpha_{\text{s}}\right)\mu_{\text{ys}}\beta \lambda_{x}\mu_{\text{w}}}{\left(\left(k_{y}\lambda_{\text{w}}/\mu_{\text{w}}\right)+\sigma_{\text{s}}+\left(\mu_{\text{ys}}/1-\omega_{\text{s}}\right)\right)\left(\zeta +\mu_{\text{ms}}+\left(k_{\text{m}}\lambda_{\text{w}}/\mu_{\text{w}}\right)+\Psi_{1}+\left(\beta \lambda_{x}\mu_{x}/\left(\gamma \lambda_{\text{w}}+\mu_{\text{w}}\right)\mu_{x}\right)\right)\left(\gamma \lambda_{\text{w}}+\mu_{\text{w}}\right)\mu_{x}},\\ R_{\text{r}}=\frac{P\left(1-\alpha_{\text{r}}\right)\mu_{\text{yr}}\delta_{\text{r}}\beta \lambda_{x}\mu_{\text{w}}}{\left(\left(k_{y}\lambda_{\text{w}}/\mu_{\text{w}}\right)+\sigma_{\text{r}}+\mu_{\text{yr}}\right)\left(\mu_{\text{mr}}+\left(k_{\text{m}}\lambda_{\text{w}}/\mu_{\text{w}}\right)+\left(\delta_{\text{r}}\beta \lambda_{x}\mu_{\text{w}}/\left(\gamma \lambda_{\text{w}}+\mu_{\text{w}}\right)\mu_{x}\right)\right)\left(\gamma \lambda_{\text{w}}+\mu_{\text{w}}\right)\mu_{x}}. \end{array}$$

From equation (31), it is evident that, in a multiple-strain *P. falciparum* malaria infection, the progression of the disease depends on the reproduction number of different parasite strains. If the threshold quantity *R*_s_ > *R*_r_, the drug-sensitive parasite strains will dominate the drug-resistant strains and hence the driver of the infection. To manage the infection in this case, the patient should be given antimalarials that can eradicate the drug-sensitive parasites. Conversely, if *R*_r_ > *R*_s_, the infection is mainly driven by the drug-resistant parasite strains. In this scenario, the used antimalarial drugs should be highly efficacious and effective enough to kill both the drug-resistant and drug-sensitive parasite strains in the blood of the human host. This result is quite instrumental in improving antimalarial therapy for *P. falciparum* infections. The best antimalarials should be sufficient enough to eradicate both parasite strains within the human host.

Based on Theorem 2 in [63], we have the following lemma.

**Lemma 2 .**

The parasite-free equilibrium point *𝔼*_0_ is locally asymptotically stable if *R*_E_ < 1(*R*_s_ < 1 and *R*_r_ < 1) and unstable otherwise.

The Jacobian matrix associated with the in-host model system (7)–(14) at *𝔼*_0_ is given by

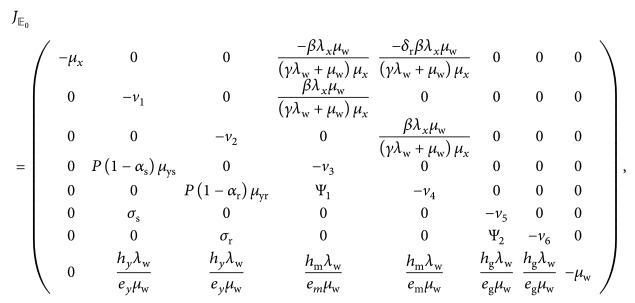
(33)where the terms *v*_1_,…, *v*_6_ are as defined in (30). It is clear from matrix (33) that the first four eigenvalues are −*μ*_*x*_ (from column 1), −*μ*_w_ (from column 8), −(*μ*_gr_+(*k*_g_ *λ*_w_/*μ*_w_))=−*v*_6_ (from column 7), and −(*η*+*μ*_gs_+(*k*_g_ *λ*_w_/*μ*_w_))=−*v*_5_ (from column 6). They are all negative. The remaining four eigenvalues are obtained from the roots of the following quartic equation:

(34) $$\begin{array}{c} P\left(\lambda \right)=\lambda^{4}+p_{1}\lambda^{3}+p_{2}\lambda^{2}+p_{3}\lambda +p_{4}, \end{array}$$

where

(35) $$\begin{array}{c} p_{1}=\left(v_{1}+v_{2}+v_{3}+v_{4}\right)>0, \end{array}$$

(36) $$\begin{array}{c} p_{2}=v_{3}v_{4}+v_{2}\left(v_{3}+v_{4}\right)+v_{1}\left(v_{2}+v_{3}+v_{4}\right)-\frac{P\beta \lambda_{x}\mu_{\text{w}}}{\left(\gamma \lambda_{\text{w}}+\mu_{\text{w}}\right)\mu_{x}}\left(\left(1-\alpha_{\text{s}}\right)\mu_{\text{ys}}-\left(1-\alpha_{\text{r}}\right)\mu_{\text{yr}}\delta_{\text{r}}\right), \end{array}$$

(37) $$\begin{array}{c} p_{3}=\frac{1}{K}\left[v_{3}\left(v_{2}v_{4}K-P\left(1-\alpha_{\text{r}}\right)\mu_{\text{yr}}\delta_{\text{r}}\beta \lambda_{x}\mu_{\text{w}}\right)\right]-\frac{1}{K}\left[P\left(1-\alpha_{\text{s}}\right)\mu_{\text{ys}}\beta \lambda_{x}\mu_{\text{w}}\left(v_{2}+v_{4}\right)+\frac{v_{1}}{K}\left[\left(v_{3}v_{4}\right)+v_{2}\left(v_{3}+v_{4}\right)\right]K-P\left(1-\alpha_{\text{r}}\right)\mu_{\text{yr}}\delta_{\text{r}}\beta \lambda_{\text{x}}\mu_{\text{w}}\right), \end{array}$$

(38) $$\begin{array}{c} p_{4}=\frac{\left(v_{2}v_{4}K-P\left(1-\alpha_{\text{r}}\right)\mu_{\text{yr}}\delta_{\text{r}}\beta \lambda_{x}\mu_{\text{w}}\right)\left(v_{1}v_{3}K-P\left(1-\alpha_{\text{s}}\right)\mu_{\text{ys}}\beta \lambda_{x}\mu_{\text{w}}\right)}{K}. \end{array}$$

Due to complexity in the coefficients of the polynomial (34), we shall rely on the Routh–Hurwitz stability criterion [64], which provides sufficient condition for the existence of the roots of the given polynomial on the left half of the plane.

**Definition 1 .**

The solutions of the quartic equation (34) are negative or have negative real parts provided that the determinants of all Hurwitz matrices are positive [64].

Based on the Routh–Hurwitz criterion, the system of inequalities that describe the stability region *𝔼*_0_ is presented as follows:

- *p*_1_ > 0
- *p*_3_ > 0
- *p*_4_ > 0
- *p*_1_ *p*_2_ *p*_3_ > *p*_3_^2^+*p*_1_^2^ *p*_4_

From (35), it is clear that *p*_1_ > 0. Upon simplifying *p*_2_ in (36), we obtain

(39) $$\begin{array}{c} p_{2}=v_{3}v_{4}+v_{2}v_{3}+v_{1}v_{2}+v_{1}v_{4}+\left(v_{1}v_{3}+\frac{\lambda_{x}\mu_{\text{w}}\beta B_{1}}{K}\right)+\left(v_{2}v_{4}+\frac{\lambda_{x}\mu_{\text{w}}\delta_{\text{r}}\beta B_{2}}{K}\right), \end{array}$$

where *B*_1_=−*P*(1 − *α*_s_)*μ*_ys_ and *B*_2_=−*P*(1 − *α*_r_)*μ*_yr_.

Thus,

(40) $$\begin{array}{c} p_{2}=v_{3}v_{4}+v_{2}v_{3}+v_{1}v_{2}+v_{1}v_{4}+v_{1}v_{3}\left[1-\frac{B_{1}\beta \lambda_{x}\mu_{\text{w}}}{v_{1}v_{3}K}\right]+v_{2}v_{4}\left[1-\frac{B_{2}\delta_{\text{r}}\beta \lambda_{x}\mu_{\text{w}}}{v_{2}v_{4}K}\right]\\ =\left(v_{1}+v_{3}\right)\left(v_{2}+v_{4}\right)+v_{1}v_{3}\left[1-R_{\text{s}}\right]+v_{2}v_{4}\left[1-R_{\text{r}}\right]>0,\;\text{if and only if}\;\;R_{\text{s}},R_{\text{r}}<1. \end{array}$$

Similarly, the expression for *p*_4_ can be rewritten as follows:

(41) $$\begin{array}{c} p_{4}=\left[v_{1}v_{3}+\frac{B_{1}\beta \lambda_{x}\mu_{x}}{K}\right]\left[v_{2}v_{4}+\frac{B_{2}\delta_{\text{r}}\beta \lambda_{x}\mu_{\text{w}}}{K}\right]\\ =v_{1}v_{3}\left[1+\frac{B_{1}\beta \lambda_{x}\mu_{\text{w}}}{v_{1}v_{3}K}\right]v_{2}v_{4}\left[1+\frac{B_{2}\delta_{\text{r}}\beta \lambda_{x}\mu_{\text{w}}}{v_{2}v_{4}K}\right]\\ =v_{1}v_{3}\left[1-R_{\text{s}}\right]v_{2}v_{4}\left[1-R_{\text{r}}\right]>0,\;\text{if and only if}\;\;R_{\text{s}},R_{\text{r}}<1. \end{array}$$

Lastly, upon simplifying equation (37), we obtain

(42) $$\begin{array}{c} p_{3}=v_{2}v_{3}v_{4}+v_{1}v_{3}v_{4}+v_{1}v_{2}\left(v_{3}+v_{4}\right)+\frac{\beta B_{1}\lambda_{x}\mu_{\text{w}}\left(v_{2}+v_{4}\right)}{K}+\frac{\delta_{\text{r}}\beta B_{2}\lambda_{x}\mu_{\text{w}}\left(v_{1}+v_{3}\right)}{K}\\ =v_{1}v_{2}v_{3}v_{4}\left[\frac{1}{v_{4}}\left(1+\frac{\beta B_{1}\lambda_{x}\mu_{\text{w}}}{v_{1}v_{3}K}\right)+\frac{1}{v_{2}}\left(1+\frac{\beta B_{1}\lambda_{x}\mu_{\text{w}}}{v_{1}v_{3}K}\right)+\frac{1}{v_{1}}\left(1+\frac{\delta_{\text{r}}\beta B_{2}\lambda_{x}\mu_{\text{w}}}{v_{2}v_{4}K}\right)+\frac{1}{v_{3}}\left(1+\frac{\delta_{\text{r}}\beta B_{2}\lambda_{x}\mu_{\text{w}}}{v_{2}v_{4}K}\right)\right]\\ =v_{1}v_{2}v_{3}v_{4}\left[\frac{v_{2}+v_{4}}{v_{2}v_{4}}\left(1-R_{\text{s}}\right)+\frac{v_{1}+v_{3}}{v_{1}v_{3}}\left(1-R_{\text{r}}\right)\right]\\ =v_{1}v_{3}\left(v_{2}+v_{4}\right)\left[1-R_{\text{s}}\right]+v_{2}v_{4}\left(v_{1}+v_{3}\right)\left[1-R_{\text{r}}\right]>0,\;\text{if and only if}\;\;R_{\text{s}},R_{\text{r}}<1. \end{array}$$

Since all the coefficients of the quartic equation (34) are nonnegative, all its roots are therefore negative or have negative real parts. Hence, the Jacobian matrix (33) has negative eigenvalues or eigenvalues with negative real parts if and only if the effective reproduction number *R*_E_ is less than unity. Equilibrium point *𝔼*_0_ is therefore locally asymptotically stable when *R*_E_ < 1 (when both *R*_s_ < 1 and *R*_r_ < 1). This implies that an effective antimalarial drug would cure the costrain infected human host, provided that the drug reduces the effective reproduction number to less than 1.

Lemma 2 shows that *P. falciparum* malaria can be eradicated/controlled within the human host if the initial parasite and cell populations are within the basin of attraction of the trivial equilibrium point *𝔼*_0_. To be certain to eradicate/control the infection irrespective of the initial parasite and cell populations, we need to prove the global stability of the parasite-free equilibrium point. This is presented in the following section.

### 3.3. Global Asymptotic Stability Analysis of the Parasite-Free Equilibrium Point

Following the work by Kamgong and Sallet [65], we begin by rewriting system (7)–(14) in a pseudotriangular form:

(43) $$\begin{array}{c} \left.\begin{array}{c} {\dot{X}}_{1}=D_{1}\left(X\right)\left(X-X_{1}^{*}\right)+D_{2}\left(X\right)X_{2},\\ {\dot{X}}_{2}=D_{3}\left(X\right)X_{2}, \end{array}\right\}, \end{array}$$

where *X*_1_ is a vector representing the densities of noninfective population groups (unparasitized erythrocytes and immune cells) and *X*_2_ represents the densities of infected/infective groups (infective *P. falciparum* parasites and/or infected host cells) that are responsible for disease transmissions. For purposes of clarity and simplicity to the reader, we shall represent (*X*_1_, 0) with *X*_1_ and (0, *X*_2_) with *X*_2_ in *ℝ*_+_^8^ × *ℝ*_+_^8^. We assume the existence of a parasite-free equilibrium in *φ*: *X* ^*∗*^=(*X*_1_^*∗*^, 0). Thus,

(44) $$\begin{array}{c} X=\left(X_{1},X_{2}\right),\\ X_{1}=\left(X,W\right),\\ X_{2}=\left(Y_{\text{s}},Y_{\text{r}},M_{\text{s}},M_{\text{r}},G_{\text{s}},G_{\text{r}}\right),\\ X_{1}^{*}=\left(\frac{\lambda_{x}}{\mu_{x}},\frac{\lambda_{\text{w}}}{\mu_{\text{w}}}\right). \end{array}$$

We analyze system (43) based on the assumption that it is positively invariant and dissipative in *φ*. Moreover, the subsystem ${\bar{X}}_{1}$ is globally asymptotically stable at *X*_1_^*∗*^ on the projection of *φ* on *ℝ*_+_^8^. This implies that whenever there are no infective malarial parasites, all cell populations will settle at the parasite-free equilibrium point *𝔼*_0_. Finally, *D*_2_ in (43) is a Metzler matrix that is irreducible for any *X* ∈ *φ*. We assume adequate interactions between and among different parasites and cell compartments in the model.

The matrices *D*_1_(*X*) and *D*_2_(*X*) are easily computed from subsystem ${\dot{X}}_{1}$ in (43) so that we have

(45) $$\begin{array}{c} D_{1}\left(X\right)=\left(\begin{array}{cc} -\mu_{x} & 0\\ 0 & -\mu_{\text{w}} \end{array}\right),\\ D_{2}\left(X\right)=\left(\begin{array}{cccccc} 0 & 0 & \frac{-\beta \lambda_{x}\mu_{\text{w}}}{\left(\gamma \lambda_{\text{w}}+\mu_{\text{w}}\right)\mu_{x}} & \frac{-\delta_{\text{r}}\beta \lambda_{x}\mu_{\text{w}}}{\left(\gamma \lambda_{\text{w}}+\mu_{\text{w}}\right)\mu_{x}} & 0 & 0\\ \frac{h_{y}\lambda_{\text{w}}}{e_{y}\mu_{\text{w}}} & \frac{h_{y}\lambda_{\text{w}}}{e_{y}\mu_{\text{w}}} & \frac{h_{\text{m}}\lambda_{\text{w}}}{e_{\text{m}}\mu_{\text{w}}} & \frac{h_{\text{m}}\lambda_{\text{w}}}{e_{\text{m}}\mu_{\text{w}}} & \frac{h_{\text{g}}\lambda_{\text{w}}}{e_{\text{g}}\mu_{\text{w}}} & \frac{h_{\text{g}}\lambda_{\text{w}}}{e_{\text{g}}\mu_{\text{w}}} \end{array}\right). \end{array}$$

We can easily see that the eigenvalues of matrix *D*_1_ are both real and negative (−*μ*_*x*_ < 0, −*μ*_w_ < 0). This shows that the subsystem ${\dot{X}}_{1}=D_{1}\left(X\right)\left(X-X_{1}^{*}\right)+D_{2}\left(X\right)X_{2}$ is globally asymptotically stable at the trivial equilibrium *X*_1_^*∗*^. Additionally, from subsystem ${\dot{X}}_{2}=D_{3}\left(X\right)X_{2}$, we obtain the following matrix:

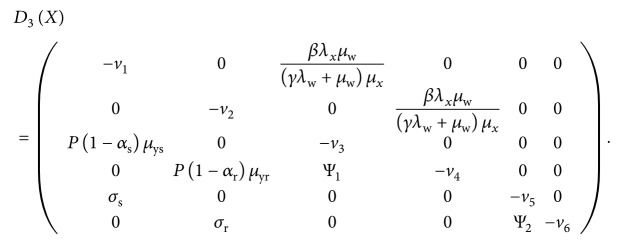
(46)


Notice that all the off-diagonal entries of *D*_3_(*X*) are nonnegative (equal to or greater than zero), showing that *D*_3_(*X*) is a Metzler matrix. To show the global stability of the parasite-free equilibrium *𝔼*_0_, we need to show that the square matrix *D*_3_(*X*) in (46) is Metzler stable. We therefore need to prove the following lemma.

**Lemma 3 .**

Let *K* be a square Metzler matrix that is block decomposed:

(47) $$\begin{array}{c} K=\left(\begin{array}{cc} K_{11} & K_{12}\\ K_{21} & K_{22} \end{array}\right), \end{array}$$

where *K*_11_ and *K*_22_ are square matrices. The matrix *K* is Metzler stable if and only if *K*_11_ and *K*_22_ − *K*_21_ *K*_11_^−1^ *K*_12_ are Metzler stable.

**Proof**

The matrix *K* in Lemma 3 refers to *D*_3_(*X*) in our case. We therefore let

(48) $$\begin{array}{c} K_{11}=\left(\begin{array}{ccc} -v_{1} & 0 & \frac{\beta \lambda_{x}\mu_{\text{w}}}{\left(\gamma \lambda_{\text{w}}+\mu_{\text{w}}\right)\mu_{x}}\\ 0 & -v_{2} & 0\\ P\left(1-\alpha_{\text{s}}\right)\mu_{\text{ys}} & 0 & -v_{3} \end{array}\right),\\ K_{12}=\left(\begin{array}{ccc} 0 & 0 & 0\\ \frac{\beta \lambda_{x}\mu_{\text{w}}}{\left(\gamma \lambda_{\text{w}}+\mu_{\text{w}}\right)\mu_{x}} & 0 & 0\\ 0 & 0 & 0 \end{array}\right),\\ K_{21}=\left(\begin{array}{ccc} 0 & P\left(1-\alpha_{\text{r}}\right)\mu_{\text{yr}} & \Psi_{1}\\ \sigma_{\text{s}} & 0 & 0\\ 0 & \sigma_{\text{r}} & 0 \end{array}\right),\\ K_{22}=\left(\begin{array}{ccc} -v_{4} & 0 & 0\\ 0 & -v_{5} & 0\\ 0 & \Psi_{2} & -v_{6} \end{array}\right). \end{array}$$

Results from analytical computations based on Maple software give

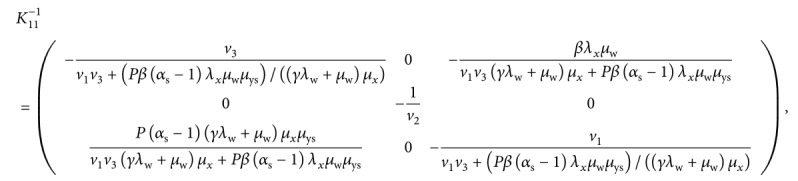
(49)

(50) $$\begin{array}{c} K_{22}-K_{21}K_{11}^{-1}K_{12}=\left(\begin{array}{ccc} -v_{4} & 0 & 0\\ 0 & -v_{5} & 0\\ 0 & \Psi_{2} & -v_{6} \end{array}\right), \end{array}$$

where *v*_4_=(*μ*_mr_+(*k*_m_ *λ*_w_/*μ*_w_)+(*δ*_r_ *βλ*_x_ *μ*_w_/(*γλ*_w_+*μ*_w_)*μ*_*x*_)), *v*_5_=(*η*+*μ*_gs_+(*k*_g_ *λ*_w_/*μ*_w_)+Ψ_2_), and *v*_6_=(*μ*_gr_+(*k*_g_ *λ*_w_/*μ*_w_)).

From equation (50), it is evident that all the diagonal elements of matrix *K*_22_ − *K*_21_ *K*_11_^−1^ *K*_12_ are negative and the rest of the elements in the matrix are nonnegative. This shows that matrix *K*_22_ − *K*_21_ *K*_11_^−1^ *K*_12_ is Metzler stable, and the parasite-free equilibrium point *𝔼*_0_ is globally asymptotically stable in the biologically feasible region φ of model system (7)–(14). Epidemiologically, the above result implies that when there is no malaria infection, different cell populations under consideration will stabilize at the parasite-free equilibrium. However, if there exists a *P. falciparum* infection, then an appropriate control in forms of effective antimalarial drugs would be necessary to clear the parasites from the human blood and restore the system to the stable parasite-free equilibrium state.

### 3.4. Coexistence of Parasite-Persistent Equilibrium Point

The existence of a nontrivial equilibrium point represents a scenario in which the *P. falciparum* parasites are present within the host and the following conditions hold: *X* ^*∗*^ > 0, *Y*_s_^*∗*^ ≥ 0, *Y*_r_^*∗*^ ≥ 0, *M*_s_^*∗*^ ≥ 0, *M*_r_^*∗*^ ≥ 0, *G*_s_^*∗*^ ≥ 0, *G*_r_^*∗*^ ≥ 0,  and *W* ^*∗*^ > 0. Upon equating the right-hand side of system (7)–(14) to zero and solving for the state variables, we obtain the parasite-persistent equilibrium point *𝔼*_1_=(*X* ^*∗*^, *Y*_s_^*∗*^, *Y*_r_^*∗*^, *M*_s_^*∗*^, *M*_r_^*∗*^, *G*_s_^*∗*^, *G*_r_^*∗*^, *W* ^*∗*^), where

(51) $$\begin{array}{c} X^{*}=\frac{\left(1+\gamma W^{*}\right)\lambda_{x}}{\beta \left(M_{\text{s}}^{*}+\delta_{\text{r}}M_{\text{r}}^{*}\right)+\left(1+\gamma W^{*}\right)\mu_{x}},\\ Y_{\text{r}}^{*}=\frac{\underline{b}+\sqrt{{\underline{b}}^{2}-4\underline{a}\underline{c}}}{-2\underline{a}}, \end{array}$$

(52) $$\begin{array}{c} \bar{a}=-a\left(\left(1-\omega_{\text{s}}\right)\sigma_{\text{s}}+\mu_{\text{ys}}\right)\left(\beta M_{\text{s}}^{*}+\beta M_{\text{r}}^{*}\delta_{\text{r}}+\left(\gamma W^{*}+1\right)\mu_{x}\right)<0, \end{array}$$

(53) $$\begin{array}{c} \bar{b}=-\beta M_{\text{s}}^{*}\left(-a\left(1-\omega_{\text{s}}\right)\lambda_{x}-\omega_{\text{s}}\sigma_{\text{s}}+\sigma_{\text{s}}+\mu_{\text{ys}}\right)-W^{*}\left(1-\omega_{\text{s}}\right)k_{y}\left(\beta M_{\text{s}}^{*}+\beta M_{\text{r}}^{*}\delta_{\text{r}}+\gamma W^{*}\mu_{x}+\mu_{x}\right), \end{array}$$

(54) $$\begin{array}{c} \bar{c}=\beta M_{\text{s}}^{*}\left(1-\omega_{\text{s}}\right)\lambda_{x}>0, \end{array}$$

(55) $$\begin{array}{c} \underline{a}=-a\left(\sigma_{2}+\mu_{\text{yr}}\right)\left(\beta M_{\text{s}}^{*}+\beta M_{\text{r}}^{*}\delta_{\text{r}}+\left(\gamma W^{*}+1\right)\mu_{x}\right)<0, \end{array}$$

(56) $$\begin{array}{c} \underline{b}=\beta M_{\text{r}}^{*}\delta_{\text{r}}\left(a\lambda_{x}-\sigma_{2}-\mu_{\text{yr}}\right)-W^{*}k_{y}\left(\beta M_{\text{s}}^{*}+\beta M_{\text{r}}^{*}\delta_{\text{r}}+\gamma W^{*}\mu_{x}+\mu_{x}\right)-\left(\sigma_{2}+\mu_{\text{yr}}\right)\left(\beta M_{\text{s}}^{*}+\gamma W^{*}\mu_{x}+\mu_{x}\right), \end{array}$$

(57) $$\begin{array}{c} \underline{c}=\beta M_{\text{r}}^{*}\delta_{\text{r}}\lambda_{x}>0, \end{array}$$

(58) $$\begin{array}{c} G_{\text{s}}^{*}=\frac{b_{1}+\sqrt{b_{1}^{2}-4a_{1}c_{1}}}{-2a_{1}},\\ G_{\text{r}}^{*}=\frac{b_{2}+\sqrt{b_{2}^{2}-4a_{2}c_{2}}}{-2a_{2}}, \end{array}$$

(59) $$\begin{array}{c} a_{1}=-a\left(\eta +\mu_{\text{g}1}+\Psi_{2}\right)<0,\\ b_{1}=a\sigma_{1}Y_{\text{s}}^{*}-W^{*}k_{\text{g}}-\eta -\mu_{\text{g}1}-\Psi_{2},\\ c_{1}=\sigma_{1}Y_{\text{s}}^{*}>0, \end{array}$$

(60) $$\begin{array}{c} a_{2}=-a\mu_{\text{g}2}<0,\\ b_{2}=aG_{1}\Psi_{2}+a\sigma_{2}Y_{\text{r}}^{*}-W^{*}k_{\text{g}}-\mu_{\text{g}2},\\ c_{2}=G_{1}\Psi_{2}+\sigma_{2}Y_{\text{r}}^{*}>0, \end{array}$$

(61) $$\begin{array}{c} M_{\text{s}}^{*}=\frac{b_{3}+\sqrt{b_{3}^{2}-4a_{3}c_{3}}}{-2a_{3}},\\ M_{\text{r}}^{*}=\frac{b_{4}+\sqrt{b_{4}^{2}-4a_{4}c_{4}}}{-2a_{4}}, \end{array}$$

(62) $$\begin{array}{c} a_{3}=-\left(a\beta M_{\text{r}}^{*}\delta_{\text{r}}\left(\zeta +\mu_{\text{ms}}+\Psi_{1}\right)+a\gamma W^{*}\mu_{\text{ms}}\mu_{x}+a\mu_{\text{ms}}\mu_{x}+a\beta P\left(1-\alpha_{\text{s}}\right)\mu_{\text{ys}}Y_{\text{s}}^{*}+\Psi_{1}\left(a\left(\gamma W^{*}+1\right)\mu_{x}+\beta \right)+a\gamma \zeta W^{*}\mu_{x}+a\beta \lambda_{x}+a\zeta \mu_{x}+\beta \zeta +\beta W^{*}k_{\text{m}}+\beta \mu_{\text{ms}}\right), \end{array}$$

(63) $$\begin{array}{c} b_{3}=-\beta M_{\text{r}}^{*}\delta_{\text{r}}\left(a\left(\alpha_{\text{s}}-1\right)PY_{\text{s}}^{*}\mu_{\text{ys}}+\zeta +W^{*}k_{\text{m}}+\mu_{\text{ms}}+\Psi_{1}\right)-\left(\alpha_{\text{s}}-1\right)\beta PY_{\text{s}}^{*}\mu_{\text{ys}}-\beta \lambda_{x}-\left(\gamma W^{*}+1\right)\mu_{x}\left(a\left(\alpha_{\text{s}}-1\right)PY_{\text{s}}^{*}\mu_{\text{ys}}+\zeta +W^{*}k_{\text{m}}+\mu_{\text{ms}}+\Psi_{1}\right), \end{array}$$

(64) $$\begin{array}{c} c_{3}=P\left(1-\alpha_{\text{s}}\right)Y_{\text{s}}^{*}\mu_{\text{ys}}\left(\beta M_{\text{r}}^{*}\delta_{\text{r}}+\left(\gamma W^{*}+1\right)\mu_{x}\right)>0, \end{array}$$

(65) $$\begin{array}{c} a_{4}=-\left(a\beta M_{\text{s}}^{*}\left(\Psi_{1}\delta_{\text{r}}+\mu_{\text{mr}}\right)+\mu_{\text{mr}}\left(a\left(\gamma W^{*}+1\right)\mu_{x}+\beta \delta_{\text{r}}\right)+a\left(1-\alpha_{\text{r}}\right)\beta PY_{2}\delta_{\text{r}}\mu_{\text{y}2}+\beta W^{*}k_{\text{m}}\delta_{\text{r}}\right), \end{array}$$

(66) $$\begin{array}{c} b_{4}=a\beta M_{\text{s}}^{*2}\Psi_{1}+M_{\text{s}}^{*}\left(-\beta \left(a\delta_{\text{r}}\lambda_{x}+\mu_{\text{mr}}\right)+a\left(1-\alpha_{\text{r}}\right)\beta PY_{2}\mu_{\text{y}2}+\Psi_{1}\left(a\left(\gamma W^{*}+1\right)\mu_{x}+\beta \delta_{\text{r}}\right)\right)+\left(1-\alpha_{\text{r}}\right)PY_{2}\mu_{\text{y}2}\left(a\left(\gamma W^{*}+1\right)\mu_{x}+\beta \delta_{\text{r}}\right)-W^{*}k_{\text{m}}\left(\beta M_{\text{s}}^{*}+\left(\gamma W^{*}+1\right)\mu_{x}\right)-\mu_{\text{mr}}\left(\gamma W^{*}+1\right)\mu_{x}, \end{array}$$

(67) $$\begin{array}{c} c_{4}=\beta M_{\text{s}}^{*2}\Psi_{1}+M_{\text{s}}^{*}\left(\left(1-\alpha_{\text{r}}\right)\beta PY_{2}\mu_{\text{y}2}+\beta \delta_{\text{r}}\lambda_{x}+\Psi_{1}\left(\gamma W^{*}+1\right)\mu_{x}\right)+\left(1-\alpha_{\text{r}}\right)PY_{2}\left(\gamma W^{*}+1\right)\mu_{x}\mu_{\text{y}2}>0, \end{array}$$

(68) $$\begin{array}{c} W^{*}=\frac{\Delta }{\mu_{\text{w}}\Delta -\left(h_{\text{g}}\left(G_{\text{s}}^{*}+G_{\text{r}}^{*}\right)+h_{\text{m}}\left(M_{\text{s}}^{*}+M_{\text{r}}^{*}\right)+h_{\text{y}}\left(Y_{\text{s}}^{*}+Y_{\text{r}}^{*}\right)\right)}, \end{array}$$

where Δ=(*e*_g_+*G*_s_^*∗*^+*G*_r_^*∗*^)(*e*_m_+*M*_s_^*∗*^+*M*_r_^*∗*^)(*e*_*y*_+*Y*_s_^*∗*^+*Y*_r_^*∗*^).

Using Descartes' “Rule of Signs” [66], it is evident that irrespective of the sign of $\bar{b}$ in (53), $\underline{b}$ in (56), *b*_1_ in (59), *b*_2_ in (60), *b*_3_ in (63), and *b*_4_ in (66), the state variables *Y*_s_^*∗*^, *Y*_r_^*∗*^, *M*_s_^*∗*^, *M*_r_^*∗*^, *G*_s_^*∗*^,  and *G*_r_^*∗*^ can only have one real positive solution. This shows that the model system (7)–(14) has a unique parasite-persistent equilibrium point *𝔼*_1_.

### 3.5. Stability of the Coexistence of Parasite-Persistent Equilibrium Point

Here, we shall prove that the coexistence of parasite-persistent equilibrium *𝔼*_1_ is locally asymptotically stable when *R*_E_ > 1  (or when  *R*_s_ > 1 and *R*_r_ > 1). We shall follow the methodology by Esteva and Vargus presented in [67], which is based on the Krasnoselskii technique [68]. This methodology requires that we prove that the linearization of system (7)–(14) about the coexistence of parasite-persistent equilibrium does not have a solution of the form

(69) $$\begin{array}{c} \bar{S}\left(t\right)={\bar{S}}_{0}e^{\xi t}, \end{array}$$

where ${\bar{S}}_{0}=\left(S_{1},S_{2},\ldots ,S_{7}\right)$, (*S*_*i*_, *ξ*) ∈ *ℂ*, and the real part of *ξ* is nonnegative (Re(*ξ*) ≥ 0). Note that *ℂ* is a set of complex numbers.

Next, we substitute a solution of the form (69) into the linearized system (7)–(14) about the coexistence of parasite-persistent equilibrium. We obtain

(70) $$\begin{array}{c} \left.\begin{array}{c} \xi S_{1}=-\left(\frac{\beta M_{\text{s}}}{1+\gamma W}+\frac{k_{\text{y}}W}{1+\gamma W}+\frac{\mu_{\text{ys}}}{1-\omega_{\text{s}}}+\sigma_{\text{s}}\right)\;S_{1}-\frac{\beta M_{\text{s}}}{1+\gamma W}S_{2}+\frac{\beta \left(C^{*}-Y_{\text{s}}-Y_{\text{r}}\right)}{1+\gamma W}S_{3},\\ \xi S_{2}=-\frac{\delta_{\text{r}}\beta M_{\text{r}}}{1+\gamma W}S_{1}-\left(\frac{\delta_{\text{r}}\beta M_{\text{r}}}{1+\gamma W}+\frac{k_{\text{y}}W}{1+aY_{\text{r}}}+\mu_{\text{yr}}+\sigma_{\text{r}}\right)S_{2}+\frac{\delta_{\text{r}}\beta \left(C^{*}-Y_{\text{s}}-Y_{\text{r}}\right)}{1+\gamma W}S_{4},\\ \xi S_{3}=-\left(\frac{\beta M_{\text{s}}}{1+\gamma W}+P\left(1-\alpha_{\text{s}}\right)\mu_{\text{ys}}\right)S_{1}+\frac{\beta M_{\text{s}}}{1+\gamma W}S_{2}-\left(\frac{k_{\text{m}}W}{1+aM_{\text{s}}}+\frac{\beta \left(C^{*}-Y_{\text{s}}-Y_{\text{r}}\right)}{1+\gamma W}+k_{1}\right)S_{3},\\ \xi S_{4}=\Psi_{1}S_{3}+\left(\frac{\delta_{\text{r}}\beta M_{\text{r}}}{1+\gamma W}+P\left(1-\alpha_{\text{r}}\right)\mu_{\text{yr}}\right)S_{2}-\left(\frac{\delta_{\text{r}}\beta \left(C^{*}-Y_{\text{s}}-Y_{\text{r}}\right)}{1+\gamma W}+\frac{k_{\text{m}}W}{1+aM_{\text{r}}}+\mu_{\text{mr}}\right)S_{4}+\frac{\delta_{\text{r}}\beta M_{\text{r}}}{1+\gamma W}S_{1},\\ \xi S_{5}=\sigma_{\text{s}}S_{1}-\left(\frac{k_{\text{g}}W}{1+aG_{\text{s}}}+k_{2}\right)S_{5},\\ \xi S_{6}=\sigma_{\text{r}}S_{2}+\Psi_{2}S_{4}-\left(\frac{k_{\text{g}}W}{1+aG_{\text{r}}}+\mu_{\text{gr}}\right)S_{5},\\ \xi S_{7}=\lambda +\left(\frac{h_{\text{g}}\left(G_{\text{s}}+G_{\text{r}}\right)}{G_{\text{s}}+G_{\text{r}}+e_{\text{g}}}+\frac{h_{\text{y}}\left(Y_{\text{s}}+Y_{\text{r}}\right)}{Y_{\text{s}}+Y_{\text{r}}+e_{\text{y}}}+\frac{h_{\text{m}}\left(M_{\text{s}}+M_{\text{r}}\right)}{M_{\text{s}}+M_{\text{r}}+e_{\text{m}}}\right)S_{7}-\mu_{\text{w}}S_{7}, \end{array}\right\} \end{array}$$

where (*C* ^*∗*^ − *Y*_s_ − *Y*_r_)=*X*, *k*_1_=(Ψ_1_+*μ*_ms_+*ζ*), and *k*_2_=(Ψ_2_+*μ*_gs_+*η*).

Upon simplifying the equations in (70), we obtain

(71) $$\begin{array}{c} \left[1+\frac{\left(1+\gamma W\right)\left(1+aY_{\text{s}}\right)\left(1-\omega_{\text{s}}\right)}{\Delta_{1}}\xi \right]S_{1}=\frac{\left(1+\gamma W\right)\left(1+aY_{\text{s}}\right)\left(1-\omega_{\text{s}}\right)}{\Delta_{1}}\left(-\frac{\beta M_{\text{s}}}{1+\gamma W}S_{2}+\frac{\beta \left(C^{*}-Y_{\text{s}}-Y_{\text{r}}\right)}{1+\gamma W}S_{3}\right),\\ \left[1+\frac{\xi \left(1+\gamma W\right)\left(1+aY_{\text{r}}\right)}{\Delta_{2}}\right]S_{2}=\frac{\left(1+\gamma W\right)\left(1+aY_{\text{r}}\right)}{\Delta_{2}}\left(-\frac{\delta_{\text{r}}\beta M_{\text{r}}}{1+\gamma W}S_{1}+\frac{\delta_{\text{r}}\beta \left(C^{*}-Y_{\text{s}}-Y_{\text{r}}\right)}{1+\gamma W}S_{4}\right),\\ \left[1+\frac{\left(1+\gamma W\right)\left(1+aM_{\text{s}}\right)}{\Delta_{3}}\xi \right]S_{3}=\frac{\left(1+\gamma W\right)\left(1+aM_{\text{s}}\right)}{\Delta_{3}}\left(\frac{\beta M_{\text{s}}}{1+\gamma W}+P\left(1-\alpha_{\text{s}}\right)\mu_{\text{ys}}S_{1}+\frac{\beta M_{\text{s}}}{1+\gamma W}S_{2}\right),\\ \left[1+\frac{\xi \left(1+\gamma W\right)\left(1+aM_{\text{r}}\right)}{\Delta_{4}}\right]S_{4}=\frac{\left(1+\gamma W\right)\left(1+aM_{\text{r}}\right)}{\Delta_{4}}\left(\Psi_{1}S_{3}+\left(\frac{\delta_{\text{r}}\beta M_{\text{r}}}{1+\gamma W}+P\left(1-\alpha_{\text{r}}\right)\mu_{\text{yr}}\right)S_{2}+\frac{\delta_{\text{r}}\beta M_{\text{r}}}{1+\gamma W}S_{4}\right),\\ \left[1+\frac{\left(1+aG_{\text{s}}\right)}{k_{\text{g}}W+k_{2}}\xi \right]S_{5}=\frac{\sigma_{\text{s}}\left(1+aG_{\text{s}}\right)}{k_{\text{g}}W+k_{2}}S_{1},\\ \left[1+\frac{\left(1+aG_{\text{r}}\right)}{k_{\text{g}}W+\mu_{\text{gr}}}\xi \right]S_{6}=\frac{\left(1+aG_{\text{r}}\right)}{k_{\text{g}}W+\mu_{\text{gr}}}\left\{\sigma_{\text{r}}S_{2}+\Psi_{2}S_{4}\right\},\\ \left[1+\frac{1}{\mu_{\text{w}}}\xi \right]S_{7}=\frac{\lambda_{\text{w}}}{\mu_{\text{w}}}+\frac{W}{\mu_{\text{w}}}\left(\frac{h_{\text{g}}\left(S_{5}+S_{6}\right)}{G_{\text{s}}+G_{\text{r}}+e_{\text{g}}}+\frac{h_{\text{y}}\left(S_{1}+S_{2}\right)}{Y_{\text{s}}+Y_{\text{r}}+e_{\text{y}}}+\frac{h_{\text{m}}\left(S_{3}+S_{4}\right)}{M_{\text{s}}+M_{\text{r}}+e_{\text{m}}}\right), \end{array}$$

where

(72) $$\begin{array}{c} \Delta_{1}=\beta M_{\text{s}}\left(1+aY_{\text{s}}\right)\left(1-\omega_{\text{s}}\right)+k_{\text{y}}W\left(1-\omega_{\text{s}}\right)\left(1+\gamma W\right)+\mu_{\text{ys}}\left(1+aY_{\text{s}}\right)\left(1+\gamma W\right)+\sigma_{\text{s}}\left(1+aY_{\text{s}}\right)\left(1-\omega_{\text{s}}\right)\left(1+\gamma W\right),\\ \Delta_{2}=\delta_{\text{r}}\beta M_{\text{r}}\left(1+aY_{\text{r}}\right)+k_{\text{y}}W\left(1+\gamma W\right)+\left(\mu_{\text{yr}}+\sigma_{\text{r}}\right)\left(1+aY_{\text{r}}\right)\left(1+\gamma W\right),\\ \Delta_{3}=\left(1+aM_{\text{s}}\right)\left(\beta \left(C^{*}-Y_{\text{s}}-Y_{\text{r}}\right)\right)+k_{\text{m}}W\left(1+\gamma W\right)+k_{1}\left(1+aM_{\text{s}}\right)\left(1+\gamma W\right),\\ \Delta_{4}=\left(1+aM_{\text{r}}\right)\left(\delta_{\text{r}}\beta \left(C^{*}-Y_{\text{s}}-Y_{\text{r}}\right)\right)+k_{\text{m}}W\left(1+\gamma W\right)+\mu_{\text{mr}}\left(1+aM_{\text{r}}\right)\left(1+\gamma W\right). \end{array}$$

Separating the negative terms, we obtain the following system:

(73) $$\begin{array}{c} \left[1+F_{j}\left(\xi \right)\right]S_{j}={\left(H\bar{S}\right)}_{j},\;\text{for}\;\;j=1,2,\ldots ,7, \end{array}$$

where

(74) $$\begin{array}{c} \left.\begin{array}{c} F_{1}\left(\xi \right)=\frac{\left(1+\gamma W\right)\left(1+aY_{\text{s}}\right)\left(1-\omega_{\text{s}}\right)}{\Delta_{1}}\xi ,\\ F_{2}\left(\xi \right)=\frac{\xi \left(1+\gamma W\right)\left(1+aY_{\text{r}}\right)}{\Delta_{2}},\\ F_{3}\left(\xi \right)=\frac{\left(1+\gamma W\right)\left(1+aM_{\text{s}}\right)}{\Delta_{3}}\xi ,\\ F_{4}\left(\xi \right)=\frac{\xi \left(1+\gamma W\right)\left(1+aM_{\text{r}}\right)}{\Delta_{4}},\\ F_{5}\left(\xi \right)=\frac{\left(1+aG_{\text{s}}\right)}{k_{\text{g}}W+k_{2}}\xi ,\\ F_{6}\left(\xi \right)=\frac{\left(1+aG_{\text{r}}\right)}{k_{\text{g}}W+\mu_{\text{gr}}}\xi ,\\ F_{7}\left(\xi \right)=\frac{1}{\mu_{\text{w}}}\xi , \end{array}\right\} \end{array}$$

with

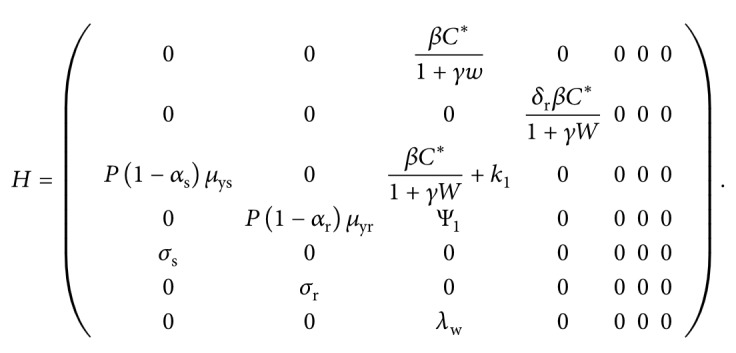
(75)


Note that *X* ^*∗*^=*C* ^*∗*^ − *Y*_s_^*∗*^ − *Y*_r_^*∗*^ and all the elements in the square matrix *H* are nonnegative. The coordinates of *𝔼*_1_ are all positive, and the *j* ^*th*^ coordinate of the vector $H\left(\bar{S}\right)$ is described by the notation $H{\left(\bar{S}\right)}_{j}$ for *j*=1,…, 7. Additionally, the equilibrium *𝔼*_1_=(*Y*_s_^*∗*^, *Y*_r_^*∗*^, *M*_s_^*∗*^, *M*_r_^*∗*^, *G*_s_^*∗*^, *G*_r_^*∗*^, *W* ^*∗*^) satisfies *𝔼*_1_=*H𝔼*_1_. If we assume, for example, that system (73) has a solution of the form $\bar{S}$, then there exists a small positive real number *ϵ*, such that $\left|\bar{S}\right|\leq \epsilon {𝔼}_{1}$, where $\left|\bar{S}\right|=\left(\left|S_{1}\right|,\left|S_{2}\right|,\ldots ,\left|S_{7}\right|\right)$. Note also that |.| is a norm in the field of complex numbers.

Next, we show that Re(*ξ*) < 0. To do so, we apply proof by contradiction. We let *ξ*=0 and *ξ* ≠ 0. For the case when *ξ*=0, the determinant (∇) of (70) is given by

(76) $$\begin{array}{c} \nabla =\frac{v_{5}v_{6}\mu_{\text{w}}\left\{v_{2}v_{4}\left(\gamma \lambda_{x}+\mu_{\text{w}}\right)\mu_{x}+P\beta \left(1-\alpha_{\text{r}}\right)\lambda_{x}\mu_{\text{w}}\mu_{\text{yr}}\right\}\left\{v_{1}v_{3}\left(\gamma \lambda_{x}+\mu_{\text{w}}\right)\mu_{x}+P\beta \left(1-\alpha_{\text{s}}\right)\lambda_{x}\mu_{\text{w}}\mu_{\text{ys}}\right\}}{{\left(\gamma \lambda_{x}+\mu_{\text{w}}\right)}^{2}\mu_{x}^{2}}, \end{array}$$

where the positive terms *v*_1_,…, *v*_6_ are as defined in matrix (29).

It is clear that the above determinant is nonnegative (∇>0). Consequently, the system (70) can only have the trivial solution $\bar{S}=\left(0,0,0,0,0,0,\lambda_{\text{w}}/\mu_{\text{w}}\right)$. On the contrary, for *ξ* ≠ 0, we assume Re(*ξ*) ≥ 0 and define *F*(*ξ*)=min|1+*F*_*j*_(*ξ*)|, *j*=1,2,…, 7. This implies that *F*(*ξ*) > 1 and *ϵ*/*F*(*ξ*) < *ϵ*. The minimality of *ϵ* means that $\left|\bar{S}\right|>\epsilon /F\left(\xi \right){𝔼}_{1}$. While considering the nonnegativity property of *H*, if we assume the norms on the two sides of (73), we shall have

(77) $$\begin{array}{c} F\left(\xi \right)\left|\bar{S}\right|\leq H\left|\bar{S}\right|\leq \varepsilon HE_{1}=\varepsilon E_{1}. \end{array}$$

This implies that $\left|\bar{S}\right|\leq \epsilon /F\left(\xi \right){𝔼}_{1}\leq \epsilon {𝔼}_{1}$, which is a contradiction. Therefore, Re(*ξ*) < 0 and *𝔼*_1_ is locally asymptotically stable when *R*_*E*_ > 1.

## 4. Numerical Simulations

### 4.1. Boundary Equilibrium Points

In this section, we show by means of numerical simulation the existence and stability of a positive parasite-persistent equilibrium point that involves only one of the parasite strains under study.

#### 4.1.1. Drug-Sensitive-Only Persistent Equilibrium Point *E*_s_

This is an equilibrium point where only the drug-sensitive parasite strains are present in the infected human host. That is, the populations *Y*_r_=*M*_r_=*G*_r_=0. This steady state is only feasible if no resistant parasites emerge from infected red blood cells and the use of antimalarial treatment does not lead to resistance development; that is, Ψ_1_=Ψ_2_=0. The original model (7)–(14) is thus reduced to

(78) $$\begin{array}{c} \left.\begin{array}{c} \frac{dX}{dt}=\lambda_{x}-\mu_{x}X-\frac{\beta XM_{\text{s}}}{1+\gamma W},\\ \frac{dY_{\text{s}}}{dt}=\frac{\beta XM_{\text{s}}}{1+\gamma W}-\frac{k_{\text{y}}Y_{\text{s}}W}{1+aY_{\text{s}}}-\frac{1}{1-\omega_{\text{s}}}\mu_{\text{ys}}Y_{\text{s}}-\sigma_{\text{s}}Y_{\text{s}},\\ \frac{dM_{\text{s}}}{dt}=\left(1-\alpha_{\text{s}}\right)P\mu_{\text{ys}}Y_{\text{s}}-\frac{\beta M_{\text{s}}X}{1+\gamma W}-\frac{k_{\text{m}}M_{\text{s}}W}{1+aM_{\text{s}}}-\left(\mu_{\text{ms}}+\zeta \right)M_{\text{s}},\\ \frac{dG_{\text{s}}}{dt}=\sigma_{\text{s}}Y_{\text{s}}-\frac{k_{\text{g}}WG_{\text{s}}}{1+aG_{\text{s}}}-\left(\mu_{\text{gs}}+\eta \right)G_{\text{s}},\\ \frac{dW}{dt}=\lambda_{\text{w}}+\left\{\frac{h_{\text{g}}\left(G_{\text{s}}\right)}{G_{\text{s}}+e_{\text{g}}}+\frac{h_{\text{y}}\left(Y_{\text{s}}\right)}{Y_{\text{s}}+e_{\text{y}}}+\frac{h_{\text{m}}\left(M_{\text{s}}\right)}{M_{\text{s}}+e_{\text{m}}}\right\}W-\mu_{\text{w}}W. \end{array}\right\} \end{array}$$

**Figure 2:**
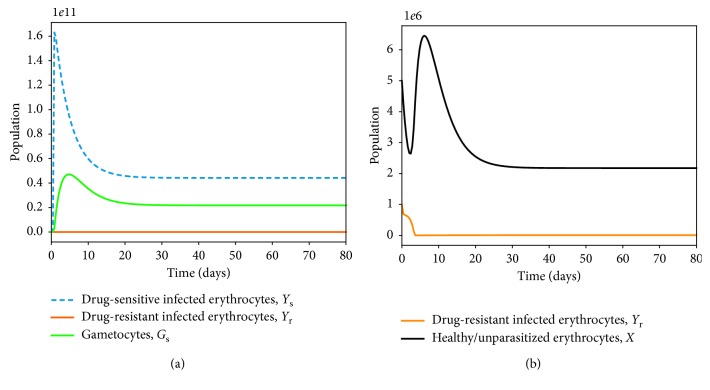
Simulations of model system (11)–(18) showing the existence of drug-sensitive-only equilibrium point. All parameter values are as presented in Table 3.

Numerically, this equilibrium point is illustrated, as shown in Figure 2.

#### 4.1.2. Drug-Resistant-Only Persistent Equilibrium Point *E*_r_

**Figure 3:**
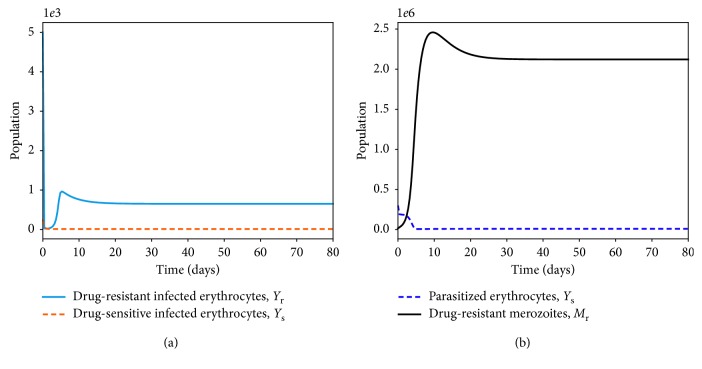
Simulations of model system (11)–(18) showing the existence of drug-resistant-only equilibrium point. All parameter values are as presented in Table 3.

In this case, the population of the drug-sensitive parasite strains declines to zero as the density of the resistant strains grows and stabilizes at an optimal population size. This is also illustrated numerically, as shown in Figure 3.

### 4.2. Within-Host Competition between Parasite Strains

**Figure 4:**
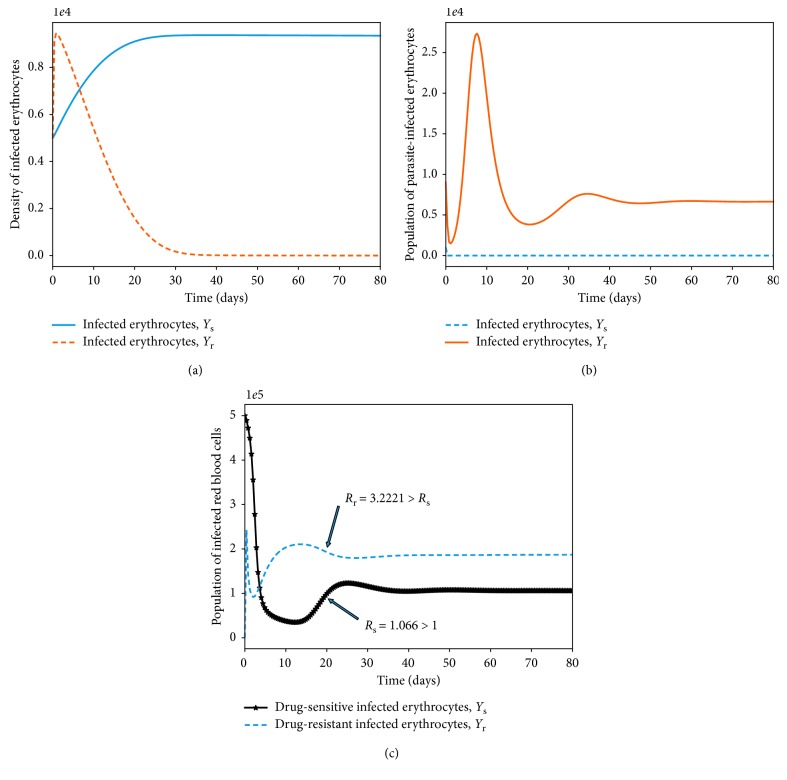
Simulations of model system (11)–(18). The figures show the dynamics of drug-sensitive and drug-resistant infected red blood cells under different conditions of the threshold values *R*_s_ and *R*_r_. In Figure 4(a), *R*_s_ > *R*_r_. In Figure 4(b), *R*_r_ > *R*_s_, Ψ_1_=0 and all other parameter values are as presented in Table 3.

We investigate the competitive exclusion principle by simulating the model system (7)–(14) under different values of the threshold quantities *R*_s_ and *R*_r_ in (31). Model (7)–(14) is simulated so that *R*_s_=4.022 and *R*_r_=0.3131, and we achieve a convergence to the drug-sensitive-only endemic equilibrium point *E*_s_, as shown in Figure 4(a). Again, using the parameter values in Table 3 with Ψ_1_=0.9 and (*R*_s_=0.022, *R*_r_=3.0098), the solutions of *Y*_s_ and *Y*_r_ converge to the drug-resistant-only endemic equilibrium point *E*_r_ (Figure 4(b)).

**Figure 5:**
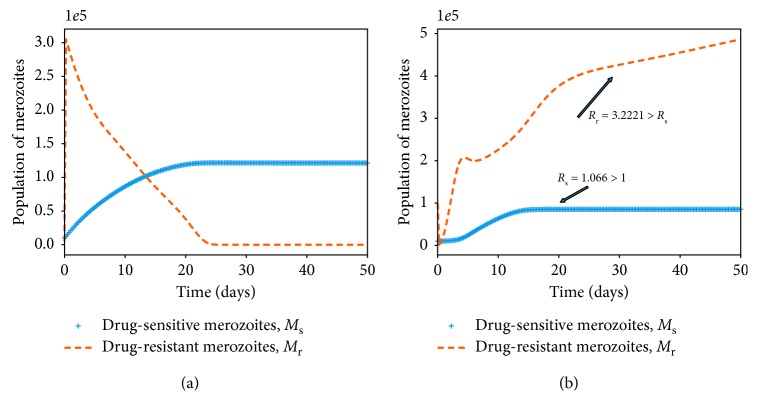
Simulations of model system (11)–(18). The figures show the dynamics of the merozoites under different conditions of the threshold values *R*_s_ and *R*_r_. Competitive exclusion among the parasite strains is shown in (a). In (b), both parasite strains coexists and *R*_r_ > *R*_s_, Ψ_1_=0. Other parameter values are available in Table 3.

Provided that both *R*_s_ and *R*_r_ are greater than 1 (as shown in Figure 4(c)), the parasite-infected red blood cells remain persistent in the host. This implies that the merozoites (both drug-sensitive and drug-resistant) continue to multiply in the absence of antimalarial therapy, *ω*_s_=0, or in the presence of ineffective antimalarial drugs. Similar results are observed in the dynamics of merozoites (*M*_s_ and *M*_r_), as shown in Figure 5. It should be noted that the dominant merozoite strains are likely to drive the infection under these conditions. As the density of one strain increases, the population of the other strain is likely to decrease due to a phenomenon known as competitive exclusion principle. The most fit parasite strain survives as the weaker competitor dies out, as shown in Figure 5(a). Both drug-sensitive and drug-resistant merozoites would remain persistent if poor-quality antimalarial drugs are administered to *P. falciparum* malaria patients. Thus, in the absence of efficacious antimalarial drugs like ACTs with the potential to eradicate resistant merozoites, we are likely to experience an exponential growth in the density of drug-resistant merozoites, as displayed in Figure 5(b). This may lead to severe malaria and eventual death of the patient.

**Figure 6:**
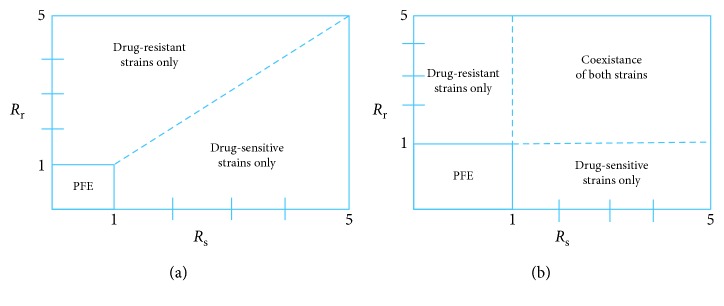
Bifurcation diagrams showing competitive exclusion (a) and coexistence equilibrium (b) for the drug-sensitive and drug-resistant *P. falciparum* parasite strains under different values of threshold quantities *R*_s_ and *R*_r_. Both parasite strains coexists when *R*_*s*_ > 1 and *R*_r_ > 1 (see part (b)).

The bifurcation analysis of both scenarios is presented in Figure 6 (with and without competition between the parasite strains). When there is competition between the parasite strains, as shown in Figure 6(a), we observe that the strain with a higher threshold quantity *R*_0_ would exclude the other strain. A decrease in the population of the drug-sensitive strain would pave way for a surge in the population of the drug-resistant strains, and vice versa. This is despite the fact that some drug-resistant strains emerge from the drug-sensitive strains as a result of mutation [77]. In Figure 6(b), we observe coexistence of the strains that do not compete with each other. Like the resistance strains, the sensitive strains are only present when their threshold quantity, *R*_s_, is greater than unity. Both strains are however present when *R*_r_ > 1 and *R*_s_ > 1. Additionally, when *R*_r_ < 1 and *R*_s_ < 1, we arrive at the parasite-free equilibrium (PFE) point, as shown in Figures 6(a) and 6(b).

### 4.3. Antimalarial Drug Effects and Parasite Clearance

The effects of antimalarial drug treatment are monitored by establishing first and foremost that

(79) $$\begin{array}{c} \frac{\partial R_{s}}{\partial \omega_{s}}=-\frac{\beta \mu_{1}\mu_{2}P\left(1-\alpha_{\text{s}}\right)\mu_{\text{w}}\lambda_{x}}{{\left(1-\omega_{\text{s}}\right)}^{2}\mu_{x}\left(\gamma \lambda_{\text{w}}+\mu_{\text{w}}\right){\left(\left(k_{\text{y}}\lambda_{\text{w}}/\mu_{w}\right)+\left(\mu_{2}/\left(1-\omega_{1}\right)\right)+\sigma_{\text{s}}\right)}^{2}\left(\zeta +\left(k_{\text{m}}\lambda_{\text{w}}/\mu_{\text{w}}\right)+\mu_{\text{ms}}+\left(\beta \lambda_{x}\mu_{x}^{2}/\left(\gamma \lambda_{\text{w}}+\mu_{\text{w}}\right)\right)+\Psi_{1}\right)}<0. \end{array}$$

**Figure 7:**
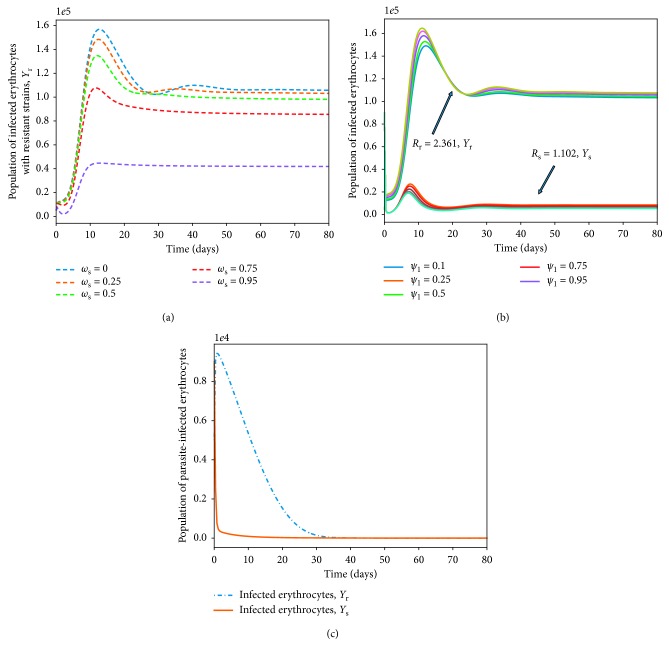
The effect of varying the efficacy of antimalarial drug used *ω*_s_ and the rate of development of resistance by the drug-sensitive merozoites Ψ_1_, on the density of infected erythrocytes (*Y*_s_, *Y*_r_). The value of *ω*_s_ ranges from 0 to 1. The rest of the parameter values are available in Table 3. Figure (c) shows that in the absence of highly effective ACTs, drug-resistant parasite would take a longer time to eradicate.

Thus, *R*_s_ is a decreasing function of *ω*_s_ (the efficacy of the antimalarial drug used). Therefore, using a highly efficient antimalarial drug could lead to a scenario where *R*_s_ < 1 and *R*_r_ < 1 (disease-free state shown in Figure 7(c)). In Figure 7(a), model system (7)–(14) is simulated by varying the efficacy of the antimalarial drug *ω*_s_ and other model parameters chosen such that *R*_r_=3.221 and *R*_s_=2.221. The higher the efficacy of the used antimalarial, the lower the density of infected erythrocytes. Thus, governments and ministry of health officers should only roll out or permit the administration of antimalarials or ACTs that can eradicate (totally) both the drug-resistant and the drug-sensitive strains of *P. falciparum* parasites.

The rate of development of resistance by the drug-sensitive merozoites, Ψ_1_, is shown to have very minimal impact on the dynamics of infected red blood cells *Y*_r_ as long as *R*_s_ > 1 and *R*_r_ > 1 (Figure 7(b)). Nevertheless, analytical results indicate that the higher the rate of development of resistance, the lower the severity of future malaria infections. This is presented as

(80) $$\begin{array}{c} \frac{\partial R_{\text{s}}}{\partial \Psi_{1}}=-\frac{\beta \mu_{1}P\left(1-\alpha_{\text{s}}\right)\mu_{\text{w}}\lambda_{x}}{\mu_{x}\left(\gamma \lambda_{\text{w}}+\mu_{\text{w}}\right)\left(\left(k_{\text{y}}\lambda_{\text{w}}/\mu_{\text{w}}\right)+\left(\mu_{2}/\left(1-\omega_{1}\right)\right)+\sigma_{\text{s}}\right){\left(\zeta +\left(k_{\text{m}}\lambda_{\text{w}}/\mu_{\text{w}}\right)+\mu_{\text{ms}}+\left(\beta \lambda_{x}\mu_{x}^{2}/\left(\gamma \lambda_{\text{w}}+\mu_{\text{w}}\right)\right)+\Psi_{1}\right)}^{2}}<0. \end{array}$$

Other parameters that have direct negative impacts on the progression of malaria infection are the efficacy of the immune effectors, *γ*, and the rate of therapeutic elimination of drug-sensitive merozoites, *ζ*:

(81) $$\begin{array}{c} \frac{\partial R_{\text{s}}}{\partial \zeta }=-\frac{\beta \mu_{1}P\left(1-\alpha_{\text{s}}\right)\mu_{\text{w}}\lambda_{x}}{\mu_{x}\left(\gamma \lambda_{\text{w}}+\mu_{\text{w}}\right)\left(\left(k_{\text{y}}\lambda_{\text{w}}/\mu_{\text{w}}\right)+\left(\mu_{2}/\left(1-\omega_{1}\right)\right)+\sigma_{\text{s}}\right){\left(\zeta +\left(k_{\text{m}}\lambda_{\text{w}}/\mu_{\text{w}}\right)+\mu_{\text{ms}}+\left(\beta \lambda_{x}\mu_{x}^{2}/\left(\gamma \lambda_{\text{w}}+\mu_{\text{w}}\right)\right)+\Psi_{1}\right)}^{2}}<0, \end{array}$$

(82) $$\begin{array}{c} \frac{\partial R_{\text{r}}}{\partial \gamma }=-\frac{\beta \mu_{2}P\left(1-\alpha_{\text{r}}\right)\delta_{\text{r}}\lambda_{\text{w}}\mu_{\text{w}}^{3}\lambda_{x}\left(k_{\text{m}}\lambda_{\text{w}}+\mu_{\text{mr}}\mu_{\text{w}}\right)}{\mu_{x}\left(k_{\text{y}}\lambda_{\text{w}}+\mu_{\text{w}}\left(\mu_{2}+\sigma_{\text{r}}\right)\right){\left(\left(\gamma \lambda_{\text{w}}+\mu_{\text{w}}\right)\left(k_{\text{m}}\lambda_{\text{w}}+\mu_{\text{mr}}\mu_{\text{w}}\right)+\beta \delta_{\text{r}}\mu_{\text{w}}\lambda_{x}\mu_{x}^{2}\right)}^{2}}<0. \end{array}$$

**Figure 8:**
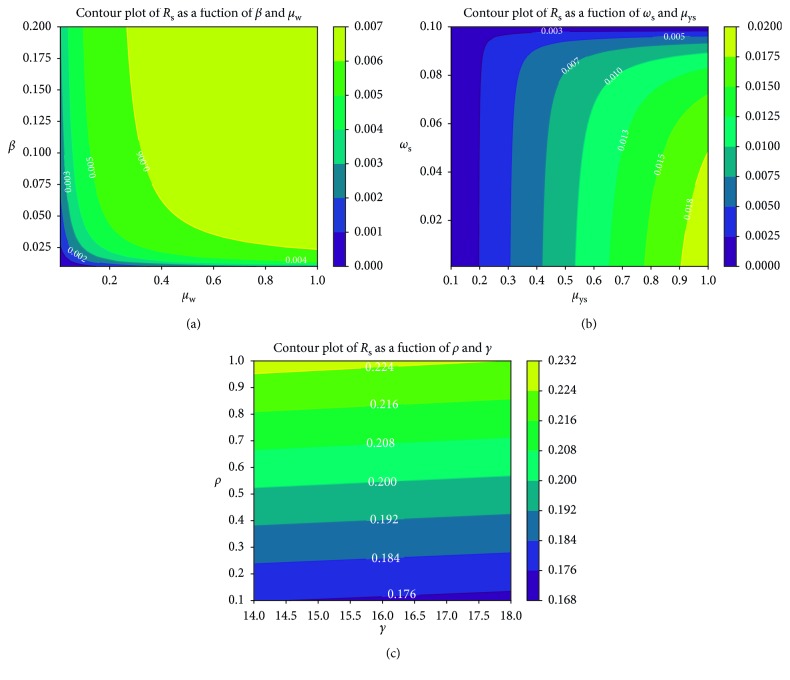
Contour plot of *R*_s_ as a function of (a) *β* and *μ*_w_, (b) *ω*_s_ and *μ*_ys_, (c) P and *γ*.

Further simulations based on contour plots (see [78] for theory on contour plots) are used to ascertain the relational effects of selected pairs of model parameters on the disease threshold quantities *R*_s_ and *R*_r_. In Figure 8(a), both *β* and *μ*_w_ increase the reproduction number due to drug-sensitive *P. falciparum* parasite strains. A direct relationship exists between the two parameters: the higher the decay rate of the immune cells, the higher the rate of infection of healthy erythrocytes.

In Figure 8(b), we observe the least increase in *R*_s_ with respect to an increase in *ω*_s_ relative to *μ*_ys_. Antimalarial therapy is shown to be very effective in reducing the severity of *P. falciparum* infection. Conversely, the number of merozoites produced per dying blood schizont, *P*, is shown in Figure 8(c) to have a very high positive impact on *R*_s_ and hence on the severity of malaria infection due to drug-sensitive parasite strains. Clinical control should target and eradicate infected red blood cells to diminish the erythrocytic cycles of infections.

**Figure 9:**
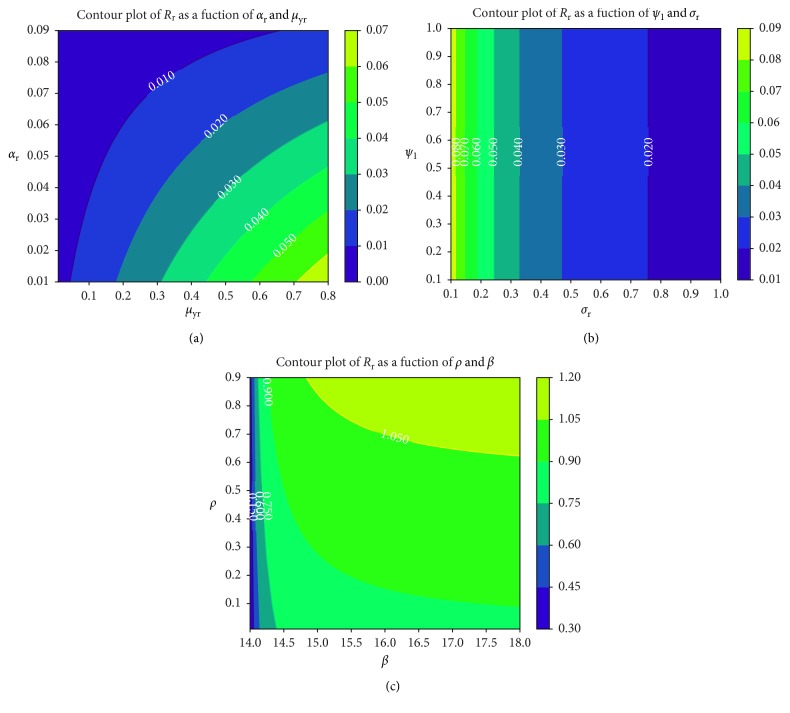
Contour plot of *R*_r_ as a function of (a) *α*_r_ and *μ*_yr_, (b) Ψ_1_ and *σ*_r_, (c) P and *β*.

We observe in Figure 9(b) that the rate at which merozoites develop resistance due to treatment failure has no resultant effects on the rate of formation of gametocytes that undergo sexual reproduction within the mosquito vector. The higher the value of *R*_r_, the higher the cost of resistance, as shown in Figure 9(a). The higher the density of drug-resistant parasite strains, the higher the level of resistance and hence the cost of disease control. Unfortunately, highly effective antimalarial drugs (such as ACTs) that can eradicate both parasite strains are slightly expensive in several *P. falciparum* malaria-endemic regions [79]. Like the parameter *P*, the parasite infection rate *β* is shown to have a direct positive effect on the threshold quantity *R*_r_ (Figure 9(c)) due to drug-resistant parasite strains. Effective antimalarials should hence target new cell infections and eliminate recrudescence (by killing already infected erythrocytes).

## 5. Effects of Multiple-Strain Infection and Fitness Cost on Parasite Clearance

**Figure 10:**
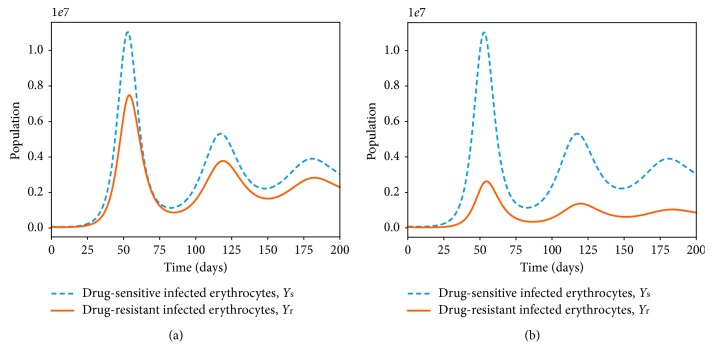
Dynamics of drug-sensitive (blue) and drug-resistant (orange) strains in a single infection (a) and in a multiple infection (b) in a naive human-host with no malaria therapy (*ω*_s_=0). The density of the resistant strain is lower than that of drug-sensitive strain for *R*_s_=2.123 > 1 and *R*_r_=1.912 > 1 in a multiple-strain *P. falciparum* infection. The rest of the parameter values are as displayed in Table 3.

Numerous studies [27, 80] have suggested the negative impacts of drug resistance on the fitness and ability of the parasite to dominate the *P. falciparum* infection. Resistance to antimalarial drugs imposes fitness cost on the drug-resistant parasite. The drug-resistant parasite strains are thought to experience impaired growth within the human host [29]. The cost of resistance is further exacerbated due to the competition between parasite strains within an infected human host. In Figure 10(a), the area under the curve for the drug-resistant strain or the number of infected erythrocytes is lower than that of the drug-sensitive strains. However, in a multiple-strain infection (Figure 10(b)), the area difference is much bigger. This implies that competition between the parasite strains within the human host could result in elimination of one of the parasite strains provided that both *R*_s_ and *R*_r_ are less than unity.

**Figure 11:**
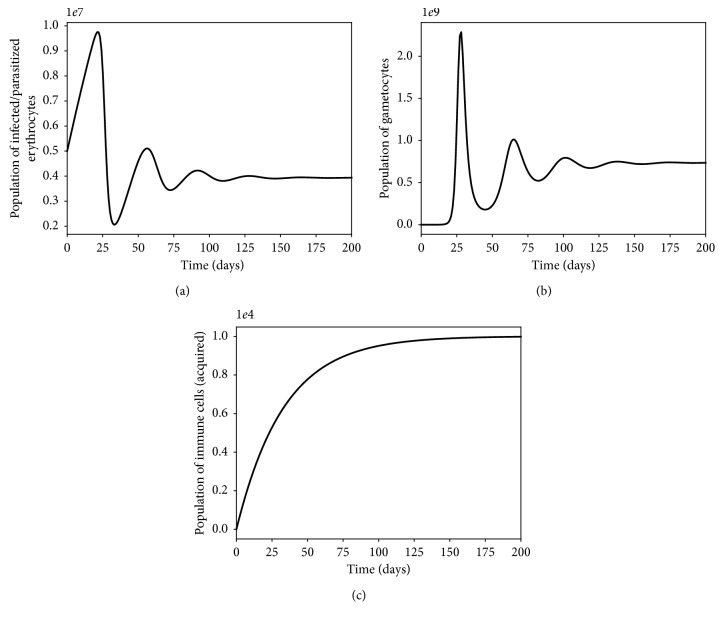
Dynamics of infected erythrocytes, gametocytes, and the immune cells with a single-strain *P. falciparum* infection. Here, we do not have preexisting immunity. The rest of the parameter values are as displayed in Table 3.

**Figure 12:**
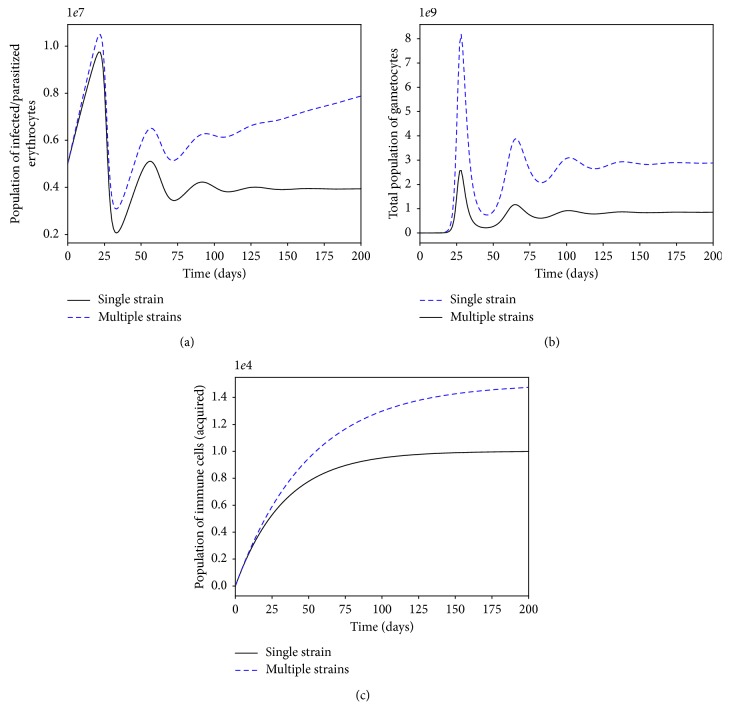
Within-human dynamics of single- and multiple-strain dynamics of infected erythrocytes, gametocytes, and the immune cells in the absence preexisting immunity and with no antimalarial treatment (*ω*_s_=0). The rest of the model parameter values are in Table 3.

The presence of multiple strains of *P. falciparum* parasites is likely to complicate and worsen the severity of malaria disease infection in humans. Figures 11 and 12 show the simulated model (7)–(14) for single- and multiple-strain infections, in the absence of preexisting immunity and antimalarial drugs. The persistence of gametocytes in Figures 11(b) and 12(b) is consistent with the actual observations of human malaria infection in the absence of antimalarial therapy [81]. Acquired immunity is shown in Figure 11(c) to increase and eventually level-off at higher levels to contain future infections.

Although the aspect of timing is key in these multiple-strain infections, we assumed here that the two strains are introduced at the same time. In the long run, it is evident in Figures 10 and 12 that the sensitive strain overtakes the resistant strain. We argue that this could be as a result of strain-specific adaptive responses that symmetrically affect the sensitive parasites.

Unlike single-strain *P. falciparum* parasite infections, data on multiple-strain infections are not readily available. Nevertheless, a multiple-strain infection (drug-sensitive and drug-resistant) as presented in this paper is biologically reasonable and consistent with that of *P. Chabaudi* described in [82].

### 5.1. Sensitivity Analysis

In this paper, the primary model output of interest for the sensitivity analysis is the infected erythrocytes (*Y*_s_, *Y*_r_). However, the effective reproduction number *R*_E_ is a threshold quantity which represents on overage the number of secondary infected erythrocytes due to merozoite invasions. We can therefore measure the sensitivity indices of the effective reproduction number of model system (7)–(14) relative to model parameters. For example, the sensitivity of *R*_E_ relative to the parameter Ψ_1_ is given by the following formulation:

(83) $$\begin{array}{c} {ϒ}_{\Psi_{1}}=\frac{\partial R_{\text{E}}}{\partial \Psi_{1}}\times \frac{\Psi_{1}}{R_{\text{E}}}. \end{array}$$

Using the parameter values in Table 3, the expressions for sensitivity for all the parameters in *R*_E_ are evaluated and presented in Table 4. The higher the numerical value of the sensitivity index (S.I), the greater the variational impact of the parameter on the disease progression. A parameter with a negative index decreases the model *R*_E_ when they are increased. On the other hand, a parameter with a positive index would generate a proportional increase in *R*_E_ when they are magnified. Results shown in Table 4 indicate that the rate of infection of healthy erythrocytes by the merozoites *β*, the density of merozoites generated from each of the bursting schizonts *P*, the efficacy of antimalarial drug used *ω*_s_, and the rate at which drug-sensitive merozoites develop resistance Ψ_1_ are the four most influential parameters, in determining the disease dynamics as presented in model system (7)–(14).

Results from sensitivity analysis emphasize the use of highly efficacious antimalarial drugs such as ACTs in malaria-endemic regions. This would mitigate the many cases of malaria in the region and further help to reduce emerging cases of parasite resistance to existing therapies. Drugs with a higher parasite clearance rate would greatly reduce resistance, which is associated with longer parasite exposure to antimalarial drugs. It is imperative, therefore, that governments and ministry of health personnel in malaria-endemic countries enforce the use of efficient antimalarial drugs that not only cure infected malaria patients but also eliminate the chance of *P. falciparum* parasites to develop resistance to existing therapy.

## 6. Conclusion

In this paper, a deterministic model of multiple-strain *P. falciparum* malaria infection has been formulated and analysed. The parasite strains are categorized as either drug-sensitive or drug-resistant. The infected erythrocytes and the malaria gametocytes are similarly grouped according to the strain of the parasite responsible for their existence. The immune cells are incorporated to reduce the invasive characteristic of the malaria merozoites. Antimalarial therapy is applied to the model but only targets red blood cells infected with drug-sensitive merozoites. Based on the next-generation matrix method, we computed the effective reproduction number *R*_E_ of the formulated model. Based on *R*_E_, it is evident that the success of *P. falciparum* infection in the presence of multiple-parasite strains is directly dependent on the ability of the individual parasite strains to drive the infection. The parasite strain with a higher threshold value, *R*_0_, is likely to dominate the infection. Prescribed antimalarial drugs should therefore be effective enough to eradicate both drug-sensitive and drug-resistant parasite strains in vivo. Linearization of the model at the parasite-free equilibrium reveals the local asymptotic stability of the trivial equilibrium point.

By rewriting the model in the pseudotriangular form, the parasite-free equilibrium is also shown to be globally asymptotically stable. Although the parasite-persistent equilibrium exists, its expression based on a single-model variable proved to be mathematically intractable. The use of antimalarial treatment may eradicate one parasite strain so that we arrive at either a drug-sensitive-only persistent equilibrium point or a drug-resistant-only persistent equilibrium point.

To assess the impacts of the different parasite strains to disease dynamics, the model is simulated for different values of the threshold quantities *R*_s_ and *R*_r_. We observed that when *R*_r_ > 1 and *R*_s_ > 1, then both parasite strains are persistent and the infection becomes severe. If *R*_r_ > 1 and *R*_s_ < 1, then the drug-sensitive parasites would decline to zero as the drug-resistant strains continue to multiply and remain persistent, increasing the severity of infections. On the other hand, if *R*_s_ > 1 and *R*_r_ < 1, then the drug-resistant parasite strains would be eradicated. Moreover, provided that the threshold quantities *R*_s_ and *R*_r_ are less than unity, the use of an efficacious antimalarial drug would help eradicate *P. falciparum* infection.

The efficacy of antimalarial drug is shown to have direct negative impact on the density of infected red blood cells. The higher the efficacy of administered antimalarial drug, the lower the population of infective merozoites and the smaller the density of infected erythrocytes. This ensures prompt recovery from malaria infections. This result is consistent with that in [72, 83]. The efficacy of antimalarial drug is however shown to have least effect on the population of drug-resistant infected erythrocytes. The rate of development of resistance by drug-sensitive parasites is also shown to drive the infection due to resistant parasite strains. Using contour plots and results from sensitivity analysis, we observe that the efficacy of antimalarial drug used *ω*_s_, the density of blood floating merozoites produced per infected erythrocyte *P*, the rate of development of resistance Ψ_1_, and the rate of infection by merozoites *β* are the most important parameters in the disease dynamics and control.

Finally, although the drug-resistant strain is shown to be less fit, the presence of both strains in the human host has a huge impact on the cost and success of antimalarial treatment. To reduce the emergence of resistant strains, it is vital that only effective antimalarial drugs are administered to patients in hospitals, especially in malaria-endemic regions. To improve malaria therapy and reduce cases of parasite resistance to existing therapy, our results call for regular and strict surveillance on antimalarial drugs in clinics and hospitals in malaria-endemic countries.

## Figures and Tables

**Table 1:** Description of the state variables of model system (11)–(18).

Variable	Description
X	Population of uninfected/unparasitized red blood cells (erythrocytes)
*Y*_s_	Population of red blood cells infected by drug-sensitive merozoites
*Y*_r_	Population of red blood cells infected by drug-resistant merozoites
*M*_s_	Population of drug-sensitive merozoites
*M*_r_	Population of drug-resistant merozoites
*G*_s_	Population of drug-sensitive gametocytes
*G*_r_	Population of drug-resistant gametocytes
*W*	Population of strain-independent immune cells

**Table 2:** Description of model parameters.

Parameter	Description
*λ*_*x*_	The rate of recruitment of red blood cells
*ω*_s_	Antimalarial treatment efficacy
*α*_s_, *α*_r_	Parasite strain-specific fitness cost
*λ*_w_	Background recruitment rate of immune cells
*e*_g_, *e*_m_, *e*_*y*_	Hill parameters in *G*_*i*_, *M*_*i*_, and *Y*_*i*_ dynamics (*i*=*s*, *r*)
*μ*_*x*_	Per capita natural mortality rate of unparasitized erythrocytes
*μ*_ys_	Natural mortality rate of drug-sensitive IRBCs
*μ*_yr_	Natural death rate of drug-resistant IRBCs
*ζ*, *η*	Rate of antimalarial eradication of merozoites and gametocytes, respectively
*μ*_ms_	Death rate of drug-sensitive merozoites
*μ*_mr_	Mortality rate of drug-resistant merozoites
*μ*_gs_	Per capita mortality rate of drug-sensitive gametocytes
*μ*_gr_	Mortality rate of drug-resistant gametocytes
*μ*_w_	Natural mortality rate of immune cells (CD8 + T cells)
*β*	The rate of infection of susceptible RBCs by blood floating merozoites
*σ*_r_, *σ*_s_	Rate of formation of gametocytes from the infected RBCs
*P*	Number of merozoites produced per dying infected RBC
*h*_*y*_	Immune cell proliferation rate due to IRBCs
*h*_m_	Immune cell proliferation rate due to asexual merozoites
*h*_g_	Immune cell proliferation rate due to gametocytes
*k*_y_	Phagocytosis rate of IRBCs by immune cell
*k*_m_	Phagocytosis rate of merozoites by immune cell
*k*_g_	Phagocytosis rate of gametocytes by immune cell
Ψ_1_	Rate of development of resistance by drug-sensitive merozoites
Ψ_2_	Rate of development of resistance by drug-sensitive gametocytes
*δ*_r_	Accounts for the reduced fitness of the resistant parasite strains
*γ*	Efficiency of immune effector to inhibit merozoite infection
1/*a*	Half-saturation constant for *Y*(*t*), *M*(*t*), and *G*(*t*)

**Table 3:** Baseline values and range for parameters of model (11)–(18).

Parameter	Value	Range	Units	Source
*λ*_*x*_	3 × 10^3^	(3 × 10^3^ − 3 × 10^8^)	Cells/*μl* ^−1^/day	[69]
*λ*_w_	30	(10–40)	Cells/*μl*/day	[70]
*ω*_s_	0.5	(0-1)	Unitless	Assumed
*α*_s_	0.4	(0.1–1)	Unitless	Assumed
*α*_r_	0.2	(0.01–1)	Unitless	Assumed
*e*_g_, *e*_m_, *e*_y_	10^4^	(10^3^ − 10^5^)	Unitless	[71]
*μ*_*x*_	1/120	(0.05–0.1)	day^−1^	[72]
*μ*_ys_	0.5	(0.3–0.8)	day^−1^	[73]
*μ*_yr_	0.3	(0.3–0.8)	day^−1^	Assumed
*μ*_ms_, *μ*_mr_	48	(46–50)	day^−1^	[69]
*μ*_gs_, *μ*_gr_	0.0625	(0.05–0.1)	day^−1^	[74]
*μ*_w_	0.05	(0.02–0.08)	day^−1^	[74]
*δ*_r_	0.7	(0.01–0.99)	Unitless	Assumed
*ζ*, *η*	0.5	(0-1)	day^−1^	[73]
*P*	16	(15–20)	Erythrocytes/day	[34]
*β*	6.5 × 10^−7^	4.8 × 10^−7^–6.8 × 10^−7^	Merozoites/day	[75]
*σ*_r_, *σ*_s_	0.02	(0.01–0.03)	day^−1^	[75]
*h*_*y*_, *h*_m_, *h*_g_	0.05	(0.01–0.08)	mm^−3^/day	[70]
*k*_*y*_, *k*_m_, *k*_g_	0.000001	(0.001–0.9)	day^−1^	[51]
Ψ_1_	0.2	(0.01–2.2)	day^−1^	Assumed
Ψ_2_	0.01	(0.001–0.1)	day^−1^	Assumed
*δ*_r_	0.3	(0-1)	Unitless	Assumed
*γ*	0.5	(0-1)	Immune cell/*μl*	Assumed
1/*a*	0.2	(0-1)	Unitless	[76]

**Table 4:** Sensitivity indices of *R*_E_ relative to the model parameters.

Parameter	S.I
*β*	+0.9988
*P*	+1.0000
*ω*_s_	−0.87513
*λ*_*x*_	+0.7199
*μ*_*x*_	−0.0016
*k*_*y*_	−0.02701
*σ*_s_	−0.7619
*γ*	−0.3333
*μ*_mr_	−0.433
*μ*_ms_	−0.52123
*μ*_yr_	−0.232
Ψ_1_	−0.77534
*μ*_ys_	−0.492
*λ*_w_	−0.3471
*ζ*	−0.0041
*δ*_r_	+0.0023
*k*_m_	−0.0020
*σ*_r_	−0.541872
*α*_r_	−0.1111
*α*_s_	−0.09891
*μ*_w_	0.3716

## Data Availability

All data used in this study are included in this published article.
